# A scoping review of rapid review methods

**DOI:** 10.1186/s12916-015-0465-6

**Published:** 2015-09-16

**Authors:** Andrea C. Tricco, Jesmin Antony, Wasifa Zarin, Lisa Strifler, Marco Ghassemi, John Ivory, Laure Perrier, Brian Hutton, David Moher, Sharon E. Straus

**Affiliations:** Li Ka Shing Knowledge Institute of St Michael’s Hospital, 209 Victoria Street, East Building, Room 716, Toronto, ON M5B 1 W8 Canada; Epidemiology Division, Dalla Lana School of Public Health, University of Toronto, 6th Floor, 155 College Street, Toronto, ON M5T 3 M7 Canada; Institute for Health Policy Management and Evaluation, University of Toronto, 4th Floor, 155 College Street, Toronto, ON M5T 3 M6 Canada; Ottawa Hospital Research Institute, Ottawa Methods Centre, Ottawa, ON Canada; Department of Medicine, Faculty of Medicine, University of Toronto, 27 King’s College Circle, Toronto, ON M5S 1A1 Canada

**Keywords:** Systematic review, Rapid review, Scoping review

## Abstract

**Background:**

Rapid reviews are a form of knowledge synthesis in which components of the systematic review process are simplified or omitted to produce information in a timely manner. Although numerous centers are conducting rapid reviews internationally, few studies have examined the methodological characteristics of rapid reviews. We aimed to examine articles, books, and reports that evaluated, compared, used or described rapid reviews or methods through a scoping review.

**Methods:**

MEDLINE, EMBASE, the Cochrane Library, internet websites of rapid review producers, and reference lists were searched to identify articles for inclusion. Two reviewers independently screened literature search results and abstracted data from included studies. Descriptive analysis was conducted.

**Results:**

We included 100 articles plus one companion report that were published between 1997 and 2013. The studies were categorized as 84 application papers, seven development papers, six impact papers, and four comparison papers (one was included in two categories). The rapid reviews were conducted between 1 and 12 months, predominantly in Europe (58 %) and North America (20 %). The included studies failed to report 6 % to 73 % of the specific systematic review steps examined. Fifty unique rapid review methods were identified; 16 methods occurred more than once. Streamlined methods that were used in the 82 rapid reviews included limiting the literature search to published literature (24 %) or one database (2 %), limiting inclusion criteria by date (68 %) or language (49 %), having one person screen and another verify or screen excluded studies (6 %), having one person abstract data and another verify (23 %), not conducting risk of bias/quality appraisal (7 %) or having only one reviewer conduct the quality appraisal (7 %), and presenting results as a narrative summary (78 %). Four case studies were identified that compared the results of rapid reviews to systematic reviews. Three studies found that the conclusions between rapid reviews and systematic reviews were congruent.

**Conclusions:**

Numerous rapid review approaches were identified and few were used consistently in the literature. Poor quality of reporting was observed. A prospective study comparing the results from rapid reviews to those obtained through systematic reviews is warranted.

**Electronic supplementary material:**

The online version of this article (doi:10.1186/s12916-015-0465-6) contains supplementary material, which is available to authorized users.

## Background

Systematic reviews are a useful tool for decision-makers because they can be used to interpret the results of individual studies within the context of the totality of evidence and provide the evidence-base for knowledge translation products, such as patient decision aids, clinical practice guidelines or policy briefs [1]. However, due to the high level of methodological rigour, systematic reviews take from 0.5 to 2 years to conduct [2] and require considerable skill to execute. According to the Cochrane Collaboration, all procedures including screening citations (titles and abstracts), screening full-text articles, data abstraction, and risk of bias appraisal, should be conducted by two individuals, independently [3]. In addition, technical expertise from librarians, research coordinators, content experts, and statisticians is required.

Health decision-makers (including clinicians, patients, managers, and policy-makers) often need timely access to health information. Although this information can be obtained through a systematic review, these research endeavours require enormous resources to complete and the timeframe required to conduct a systematic review may not suit the needs of some decision-makers. For example, it has been estimated that systematic reviews take, on average, 1,139 hours (range 216–2,518 hours) to complete and usually require a budget of at least $100,000 [4]. Consequently, decision-makers may be forced to rely on less robust evidence, such as expert opinion or the results of a single small study [5], leading to suboptimal decision-making.

Rapid reviews are a form of knowledge synthesis in which components of the systematic review process are simplified or omitted to produce information in a timely manner [2]. Yet rapid reviews might be susceptible to biased results as a consequence of streamlining the systematic review process [6]. Although numerous rapid review programs exist internationally [7], few studies have examined their methodology. We aimed to examine rapid review approaches, guidance, impact, and comparisons through a scoping review.

## Methods

### Definition of a rapid review

A formal definition for a rapid review does not exist. As such, we used the following working definition, ‘a rapid review is a type of knowledge synthesis in which components of the systematic review process are simplified or omitted to produce information in a short period of time’ [2].

### Protocol

A scoping review protocol was compiled using guidance from Arksey and O’Malley [8], and revised upon feedback received from the Canadian Institutes of Health Research peer review panel. It is available from the corresponding author upon request.

### Information sources and literature search

To identify potentially relevant studies for inclusion, the following electronic databases were searched: MEDLINE; EMBASE; and the Cochrane Library. Since two systematic reviews have already been published on rapid reviews [6, 7], we limited our search from 2008 until May 2013. An experienced librarian (LP) drafted the literature searches based on the previous reviews, which was refined through team discussion. The MEDLINE search strategy is presented in Additional file 1: Appendix 1 and the other searches are available from the corresponding author upon request.

Our literature search was supplemented by targeted internet searches for unpublished rapid review reports posted on the websites of producers of rapid reviews. For this search, we took a random 10 % sample of the unpublished rapid reviews available on the producers’ websites. Often only the title was available for the rapid reviews, so, we focused inclusion to the full rapid review, if available. The reference lists of relevant reviews were scanned [6, 7], as were the reference lists of all included rapid reviews.

### Inclusion criteria

Articles, papers, books, and reports were included if they evaluated, compared, used or described a rapid review according to the authors.

### Screening process

The screening criteria were established *a priori* (as outlined in our protocol) and calibrated amongst the team through a series of pilot tests. After >90 % agreement was observed, pairs of reviewers screened the literature search results independently, and discrepancies were resolved through discussion. All screening was performed using our online tool, synthesi.*sr* [9].

### Data items and data abstraction process

A data abstraction form was developed *a priori* and the draft form was calibrated amongst the team using a random sample of ten included studies. After this exercise, the data abstraction form was revised and all included studies were abstracted by two reviewers working independently. Discrepancies were resolved through discussion.

Data items included study characteristics (for example, first author, year of publication), terminology used to describe the rapid review, full citation of previous methods papers that were used to guide the rapid review design, timeframe (in months) for completing the rapid review, and operationalized steps of the rapid review, if reported. The rapid review type was categorized as an application (for example, a rapid review report), development (paper attempts to further refine the rapid review method), impact (examines the impact of rapid reviews) or comparison (compares the results of a rapid review to a systematic review). We abstracted the assessment of the rapid review approach, including accuracy of results, comprehensiveness, potential for risk of bias, timeliness, cost-effectiveness, and feasibility as reported by the publication authors. We also abstracted the skills or knowledge required to conduct the rapid review as reported by the authors.

### Synthesis

To synthesize the descriptive results, we conducted qualitative analysis using NVivo 10 [10]. Content analysis was conducted by one team member (WZ) and verified by another team member (ACT) to synthesize common methodologies used across the included rapid reviews using a framework. The framework was developed by the review team and presented in Additional file 1: Appendix 2. The framework focused on the following steps for a rapid review: literature search (number of databases and grey literature); inclusion criteria (limited by date, language, and study design); screening (title/abstract and full-text); data abstraction; risk of bias/quality appraisal; and data synthesis. In order to depict the frequency of the terms used to describe the rapid reviews, a word cloud was created using Wordle, which is software that generates ‘word clouds’ from text that the user provides and places more emphasis on words that occur with greater frequency [11].

## Results

### Literature search

**Fig. 1:**
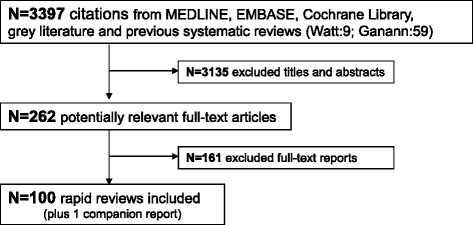
Study flow diagram

A total of 3,397 citations and 262 potentially relevant full-text papers were screened. Subsequently, 100 articles [2, 12–110] plus one companion report [111] fulfilled the eligibility criteria and were included [31] (Fig. 1). Forty-seven of the included papers were unpublished rapid reviews posted on websites [13, 24, 29, 31–36, 39, 45, 47, 50, 52–57, 62, 63, 66, 68, 70, 73–75, 77, 81–83, 86–94, 99, 100, 103, 104, 107, 109, 112].

### Rapid review characteristics and assessment

The rapid reviews were published between 1997 and 2013, and 58 were conducted in Europe, while 20 were conducted in North America (Table 1, Additional file 1: Appendix 3). The type of articles included 84 application papers (two did not report any methods), seven development papers, six impact papers, and four comparison papers; one article [20] was categorized in two categories. Ten of the rapid reviews were reported in 5 pages or less, suggesting that they were brief reports or research letters. Most of the articles (73 %) did not report the duration of conduct for the rapid review. For the minority that reported this, the duration ranged from less than 1 month to 12 months, and 18 were between 1 and 6 months. For the application articles, 74 % examined interventions, 12 % charted the frequency of literature (for example, regarding outcomes or frameworks), 5 % examined associations between exposure and disease, 5 % assessed diagnosis or screening techniques, and 2 % examined the patient experience or barriers/facilitators.

**Table 1:** Summary of study characteristics

Study characteristics	Number of rapid reviews (n = 100)^a^
Year of publication	
1997–2000	2
2001–2004	10
2005–2008	30
2009–2012	51
2013	5
Not reported	4
Continent	
Europe (including UK)	58
North America (Canada and United States)	20
Australia	15
Multiple continents	3
Asia	1
South America	1
Not reported	2
Article type^b^	
Application (82 with methods)	84
Development	7
Impact	6
Comparison	4
Topic of review	
Intervention	62 (74 %)
Frequency	10 (12 %)
Causal association	4 (5 %)
Diagnosis	4 (5 %)
Patient experience Screening	2 (2 %)
	2 (2 %)
Not applicable	16
Some methods reported	
Yes	82
No	18
Review question	
Clearly reported	81
Unclear/not reported	1
Not applicable	18

^a^100 relevant articles and one companion report (companion report not included in this table); ^b^one development article was also categorized as a comparison paper

Sixty-five articles assessed rapid review characteristics (Table 2) [2, 12, 14–22, 24, 26–30, 32, 37–39, 41–43, 45–49, 51–59, 61, 63, 64, 66, 69, 72–76, 78–80, 84, 86, 88–94, 100, 103–105, 110]. Sixty percent of the authors reported that the report was timely, 29 % believed that the method had potential risk of bias, 23 % deemed that the approach was accurate compared to a full systematic review, 8 % believed the approach was comprehensive, 5 % reported that the approach was cost-effective, and 6 % believed it was a feasible approach.

**Table 2:** Assessing the characteristics of rapid reviews compared to systematic reviews

Characteristic assessed (n = 65)^a^	Yes (%)	Limited (%)	Unknown (%)	Not reported (%)
Accuracy	15 (23 %)	5 (8 %)	3 (5 %)	42 (64 %)
Comprehensiveness	5 (8 %)	46 (71 %)	4 (6 %)	10 (15 %)
Risk of bias	19 (29 %)	19 (29 %)	3 (5 %)	24 (37 %)
Timeliness	39 (60 %)	1 (2 %)	1 (2 %)	23 (35 %)
Cost-effectiveness	3 (5 %)	0	0	62 (95 %)
Feasibility	4 (6 %)	3 (5 %)	0	58 (89 %)

^a^65 of the 100 studies reported this information

### Terminology used to describe the rapid review method

**Fig. 2:**
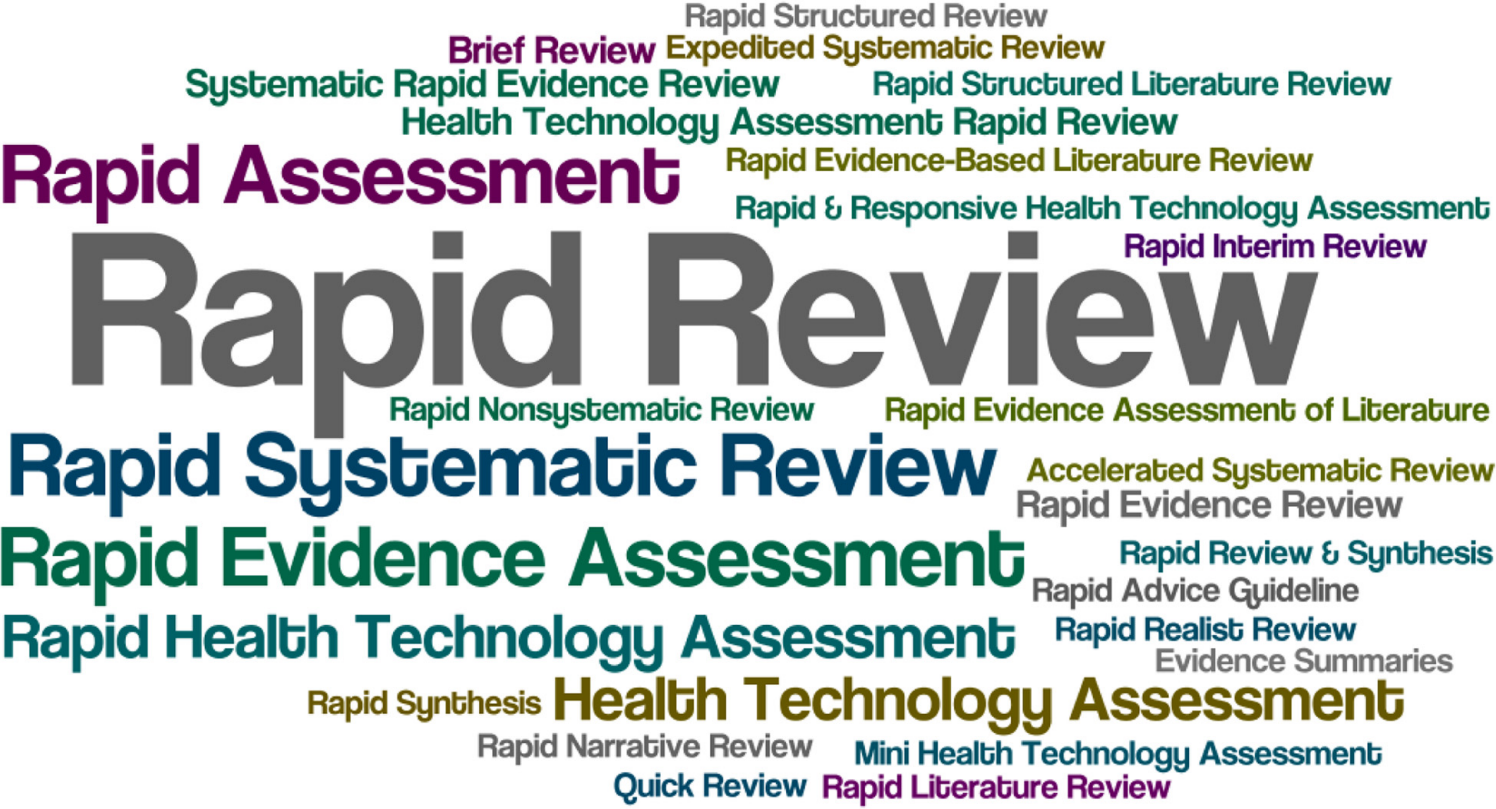
Word cloud for the frequency of terms

The most frequent term used to describe the rapid review approaches was ‘rapid review’, used in 34 of the included articles (Fig. 2). This was followed by ‘rapid evidence assessment’, which was used in 11 papers, ‘rapid systematic review’ in ten papers, and ‘health technology assessment’ or ‘rapid health technology assessment’ in six papers. All of the other terms occurred two times or less.

### Citation analysis

**Fig. 3:**
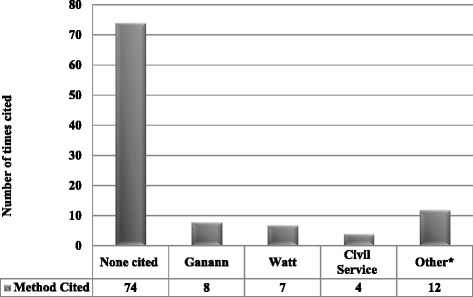
Citation analysis. *Twenty-six papers referenced another seminal paper to establish their rapid review framework

Twenty-six [2, 12, 13, 17, 20–22, 27, 28, 30, 40, 42–44, 48, 49, 61, 76, 78–80, 84, 88, 103, 105, 110] articles provided citations of previous methods papers that were used to guide the rapid review method (Fig. 3, Additional file 1: Appendix 4). The citations were Ganann and colleagues [6] (cited in eight papers), Watt and colleagues [7, 111] (cited in seven papers), a Civil Service paper [113] (cited in four papers), Ehlers and colleagues [114] (cited in one paper), Armitage and colleagues [14] (cited in one paper), and Grant and colleagues [115] (cited in one paper).

### Skills and knowledge required to conduct the rapid reviews

Thirteen [16, 32, 39, 42, 46, 48, 49, 52, 79, 84, 88, 90, 94] of the included papers reported the skills and knowledge required to conduct the rapid reviews (Table 3). These were content experts in seven articles [16, 32, 42, 48, 49, 79, 90], information specialists in five articles [39, 49, 52, 84, 88], systematic review methodologists in four papers [16, 42, 48, 79], staff experienced in conducting reviews in four papers [46, 48, 49, 84], and knowledge users in three papers [32, 79, 94].

**Table 3:** Skills required to conduct a rapid review

	Skills required^a^				
Author, year	Content experts	Information specialists	Experienced staff	Methodologists	Knowledge users
Bambra, 2010	✓			✓	
Brunton, 2013		✓			
Carr, 2011	✓				
Clark, 2003	✓				✓
Foerster, 2007		✓			
Hailey, 2009	✓			✓	
Jahangirian, 2011			✓		
Kelly, 2011	✓		✓	✓	
Konnyu, 2012	✓	✓	✓		
Low, 2006		✓			
Thigpen, 2012	✓			✓	✓
Tripney, 2011					✓
York, 2011		✓	✓		

^a^As reported by the authors

### Operationalized steps to conduct the rapid review applications

The 84 rapid review applications were categorized using our framework (Additional file 1: Appendix 2) and 50 unique methods were observed. Of these, only 16 occurred more than once; three approaches occurred five times [21, 36, 40, 44, 45, 47, 53, 54, 56, 57, 65, 75, 83, 91, 92], another two occurred four times [18, 37, 39, 64, 86, 93, 99, 107], three approaches were used three times [49, 51, 58, 61, 62, 69, 73, 76, 81], and eight approaches occurred two times [14, 16, 20, 25, 27, 30, 31, 66–68, 70, 79, 82, 96, 100, 104]. The characteristics of the rapid review approaches that occurred more than four times were analyzed (Table 4). Rapid Approach 1 had the most details reported, with 5/5 papers mentioning that it was accurate and timely (but did not report the amount of time it took to conduct their rapid review), and had limited comprehensiveness.

**Table 4:** Evaluation of rapid review approaches occurring more than four times

Rapid review approach	Author, year	Duration of review	Accuracy	Comprehensiveness	Risk of bias	Timeliness	Cost-effectiveness	Feasibility
Approach 1. Literature search: searched more than one database, limited to published sources only. Search limit: limited by both date and language. Screening: title/abstract and full-text screening performed by one reviewer only. Data abstraction: one person abstracted data, while another person verified the data risk of bias assessment; one person assessed risk of bias, while another person verified the risk of bias assessment	Blank, 2012	NR	Accurate	Limited	Potential ROB	Timely	NR	NR
	Maddern, NR	NR	Accurate	Limited	NR	Timely	NR	NR
	Maddern, NR	NR	Accurate	Limited	NR	Timely	NR	NR
	Maddern, 2008	NR	Accurate	Limited	NR	Timely	NR	NR
	Maddern, NR	NR	Accurate	Limited	NR	Timely	NR	NR
Approach 2. Literature search: used previous review(s) as starting point; searched published sources only. Search limit: no language or date limits applied. Screening: title/abstract and full-text screening performed by one reviewer only. Data abstraction: data abstraction performed by one reviewer only. Risk of bias assessment: not performed	Van de Velde, 2011	1 month	NR	NR	NR	NR	NR	NR
	Mitchell, 2011	3–4 days	Unknown accuracy	Limited	NR	Timely	Cost-effective	NR
	Government Social Research, 2007	8–12 weeks	NR	NR	NR	NR	NR	NR
	Dixon-Woods, 2012	NR	NR	NR	Potential ROB	NR	NR	NR
	Van Brabandt, 2008	NR	NR	NR	NR	NR	NR	NR
Approach 3. Literature search: searched more than one database, searched both published and grey literature. Search limit: limited by both date and language. Screening: title/abstract and full-text screening performed by one reviewer only. Data abstraction: data abstraction performed by one reviewer only. Risk of bias assessment: not performed	Foerster, 2007	NR	NR	NR	NR	NR	NR	NR
	Beck, 2012	NR	NR	NR	NR	Timely	NR	NR
	Rissel, 2012	NR	NR	NR	NR	NR	NR	NR
	ASERNIP – Surgical, 2009	NR	NR	Limited	Potential ROB	NR	NR	NR
Approach 4. Literature search: searched more than one database, searched both published and grey literature. Search limit: limited by either date or language. Screening: title/abstract and full-text screening performed by one reviewer only. Data abstraction: data abstraction performed by one reviewer only. Risk of bias assessment: not performed	Hildon, 2012	NR	NR	NR	NR	NR	NR	NR
	Jolliffe, 2008	NR	Limited accuracy	Limited	Potential ROB	timely	NR	NR
	De Laet, 2008	NR	NR	NR	NR	NR	NR	NR
	Hulstaert, 2009	NR	NR	Limited	NR	NR	NR	NR
	Moran, 2011	NR	NR	NR	NR	NR	NR	NR
Approach 5. Literature search: searched more than one database, searched both published and grey literature. Search limit: limited by date only; no language limits applied. Screening: title/abstract and full-text screening performed by one reviewer only. Data abstraction: data abstraction performed by one reviewer only. Risk of bias assessment: risk of bias assessed by one reviewer only	Phillipson, 2012	NR	NR	NR	NR	NR	NR	NR
	Geddes, 2011	NR	NR	NR	NR	NR	NR	NR
	Doran, 2013	NR	NR	Unknown	Potential ROB	NR	NR	NR
	Vlayen, 2006	NR	NR	NR	NR	NR	NR	NR
	Singh, 2006	3 weeks	NR	Limited	NR	NR	NR	NR

NR, not reported; ROB, risk of bias

Many of the steps used in the rapid reviews were not fully reported (Table 5, Additional file 1: Appendix 5). For example, 40 % (33/82) did not report whether reference lists were scanned and 67 % (55/82) did not report whether authors were contacted to obtain further material or information.

**Table 5:** Summary of rapid review streamlined approaches (n = 82 application studies)

Rapid review methods		Count (%)
General		
Duration of review		
	>6 months	3 (4 %)
	≤6 months	19 (23 %)
	Not reported	60 (73 %)
Published protocol		
	Mentioned	2 (2 %)
	Not mentioned	80 (98 %)
Review question		
	Clearly reported	81 (99 %)
	Unclear/inferred	1 (1 %)
Identifying relevant studies		
Databases searched		
	Searched more than one database	67 (82 %)
	Searched one database only	2 (2 %)
	Used a previous review(s) as starting point	8 (10 %)
	Not reported	5 (6 %)
Grey literature		
	Searched grey literature	57 (70 %)
	No grey literature search	20 (24 %)
	Not reported	5 (6 %)
Search strategy		
	Clearly reported	64 (78 %)
	Unclear	7 (9 %)
	Not reported	11 (13 %)
Scanned references		
	Yes	41 (50 %)
	No	8 (10 %)
	Not reported	33 (40 %)
Contacted authors		
	Yes	18 (22 %)
	No	9 (11 %)
	Not reported	55 (67 %)
Limits applied		
Date		
	No limit	10 (12 %)
	Limited by date	56 (68 %)
	Not reported	16 (20 %)
Language		
	No limit	14 (17 %)
	Limited by language	40 (49 %)
	Not reported	28 (34 %)
Selecting relevant studies		
Titles and abstracts		
	Two or more independent reviewers	28 (34 %)
	One reviewer and one verifier	4 (5 %)
	One reviewer only	15 (18 %)
	Done but unclear number of reviewers	20 (24 %)
	Not done	1 (1 %)
	Not reported	14 (17 %)
Full-texts		
	Two or more independent reviewers	20 (24 %)
	One reviewer and one verifier	5 (6 %)
	One reviewer only	9 (11 %)
	Done but unclear number of reviewers	23 (28 %)
	Not done	1 (1 %)
	Not reported	24 (29 %)
Data abstraction and quality appraisal		
Data abstraction		
	Two or more independent reviewers	8 (10 %)
	One reviewer and one verifier	19 (23 %)
	One reviewer only	6 (7 %)
	Done but unclear number of reviewers	30 (37 %)
	Not done	1 (1 %)
	Not reported	18 (22 %)
Quality appraisal		
	Two or more independent reviewers	14 (17 %)
	One reviewer and one verifier	11 (13 %)
	One reviewer only	6 (7 %)
	Done but unclear number of reviewers	24 (29 %)
	Not done	6 (7 %)
	Not reported	21 (26 %)
Data synthesis		
Data synthesis		
	Meta-analysis or clear reasons for not pooling results	18 (22 %)
	Narrative/descriptive summary only	64 (78 %)

**Fig. 4:**
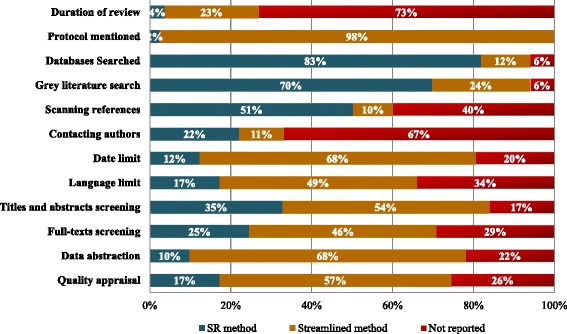
Streamlined steps used across the rapid reviews (n = 82 studies reporting this information). SR, systematic review

Streamlined methods that were used in the 82 rapid reviews included limiting the literature search to published literature (24 %) or one database (2 %), limiting inclusion criteria by date (68 %) or language (49 %), having one person screen and another verify or screen excluded studies (6 %), having one person abstract data and another verify (23 %), not conducting risk of bias/quality appraisal (7 %) or having only one reviewer conduct the quality appraisal (7 %), and presenting results as a narrative summary (78 %) (Fig. 4).

### Comparing results from rapid reviews to systematic reviews

Four studies were comparisons, providing details on differences in results between rapid reviews and systematic reviews [20, 31, 34, 106]. Cameron and colleagues identified rapid reviews from health technology assessment (HTA) organization websites and then conducted a literature search to identify systematic reviews on the same topic [31]. Eight rapid review products were identified on four different topics. However, the authors did not appraise the methodological quality of the systematic reviews, so it is unclear whether shortcuts were also taken in the included systematic reviews. The authors noted that the conclusions did not differ substantially between the rapid and systematic reviews. Corabian and colleagues compared six rapid review products (called ‘technotes’) with their final peer-reviewed publications [34]. The authors found that the conclusions differed only in 1/6 cases. Van de Velde and colleagues compared the results from their rapid review to a systematic review that was conducted by another group and published on the same topic [106]. Despite having literature searches that were conducted for the same dates, conflicting results were observed; the rapid review concluded that potato peel was effective for burns, while the systematic review concluded that potato peel was not effective for treating burns. Finally, Best and colleagues noted that two of the rapid reviews they conducted were in agreement with systematic reviews published at a later point in time on the same topic [20].

### Development papers on rapid reviews

Seven papers proposed methods to refine the rapid review approach [2, 12, 16, 20, 46, 79, 80]. Best and colleagues (1997) described their experience conducting 63 rapid reviews for decision-making beginning in 1991, through the Development and Evaluation Committee in the UK [20]. Abrami and colleagues (2010) described ways to produce brief reviews efficiently, and presented a checklist for the conduct and reporting of brief reviews [12]. Bambra and colleagues (2010) described their experience conducting nine rapid reviews for the Secretary of State for Health [16]. Jahangirian and colleagues (2011) described their experience conducting five rapid reviews for the Research into Global Healthcare Tools consortium and proposed a framework for the conduct of rapid reviews [46]. Khangura and colleagues (2012) described their approach to the conduct of 11 rapid reviews through the collaboration between the Ottawa Hospital Research Institute and the Champlain Local Health Integrated Network [2]. Thigpen and colleagues (2012) described their experience conducting rapid reviews using the 6-step Prevention Synthesis and Translation System process for the Division of Violence Prevention, National Center for Injury Prevention and Control at the Centers for Disease Control and Prevention [79]. Thomas and colleagues (2013) described their experience of conducting two rapid reviews for the UK Treasury to inform the 2006/07 Comprehensive Spending Review [80].

Guidance to streamline the rapid review process varied, yet some consistencies were observed (Table 6). For example, four papers suggested using integrated knowledge translation, in which researchers work closely with the knowledge users to complete the rapid review [2, 16, 19, 79]. Four papers suggested the use of a research question with a limited scope [12, 16, 80, 110]. Seven publications recommended streamlining the literature search [2, 12, 16, 46, 79, 80, 110] and three suggested restricting the eligibility criteria [2, 12, 80]. Two papers provided suggestions for efficiently appraising risk of bias [2, 80] and none suggested conducting a meta-analysis as part of the rapid review.

**Table 6:** Guidance provided in development papers on rapid reviews

Author, year	Overall approach to the rapid review	Question	Literature search	Screening	Data abstraction	Risk of bias	Synthesis	Dissemination/knowledge translation
Best, 1997	Use a fixed structure	Identified by purchasers and providers	Electronic databases and grey literature	Not reported	Limit the outcomes to cost-effectiveness	Not reported	Descriptive. Focus on benefits/disbenefits and costs/savings	Report provided to the committee who meets every 3 months to make decisions
Abrami, 2010	Use of a larger staff to conduct the review in a timelier manner. Use of tools to make the process more efficient	Specific research question	Updating or expanding an existing review	Use strict inclusion criteria. Only screen a random sample of results. Bypassing steps that check for inter-rater agreement	Not reported	Not reported	Descriptive only. Use of vote counting. Charting results only	Not reported
Bambra, 2010	Not reported	Limited scope	Rapid search of the literature to limited key words and databases. Restrict searches by date, accessibility, and policy relevance	Not reported	Not reported	Appraise evidence	Develop key recommendations	Refine key recommendations using a Delphi approach with end-users
Jahangirian, 2011	Incremental and iterative	Not reported	Forward citation searching and backward citation searching^a^	3-stage screening phase (filtering, sampling, and sifting)	Use graphical tools that allow the charting of the literature	Not reported	Not reported	Not reported
Khangura, 2012	Work closely with end-users using integrated knowledge translation	1–2 hours to refine question with policy-makers. Iterative process	Targeted literature searches. Includes published and unpublished literature. Focus inclusion on systematic reviews	Limited to English. Liberal accelerated^b^	Not reported	Use the level of evidence based on a modified framework established by the Cochrane Musculoskeletal Group	Descriptive synthesis only. Concise report; 1-page brief	Collaborative approach. Use feedback on previous products to improve future products
Thigpen, 2012	Work closely with end-users using integrated knowledge translation	Consult with end-user to decide on the topic	Internal and external experts engaged to focus literature search	Researchers and end-users engaged in establishing relevance	Focus on common components and key messages	Not reported	Distill the research literature	Interpretation guided by end-users to ensure relevance, understanding, and actionable knowledge. Use of 2–4-paged user-friendly briefs
Thomas, 2013	Require an experienced team in systematic reviews to conduct the rapid reviews. Prioritize rapid reviews for urgent decisions	Clearly defined. Limited scope. Limiting stakeholder involvement to provide insight into the question and protocol	Targeted searches of key databases	Limiting inclusion to English papers. Only one person screens the literature results and another screens random sample or list of excludes	Mapping study characteristics. Focusing abstraction on key interventions and specific study designs	Selecting key elements of quality appraisal tools and only appraising these	Use a framework synthesis	Not reported

^a^Forward citation searching, searching for papers that cite the included studies; backward citation searching, scanning the references of the included studies; ^b^Liberal accelerated, having a second reviewer screen the list of excluded studies.

### Articles assessing the impact and use of rapid reviews

Six papers examined the impact of rapid reviews on decision-making [41–43, 60, 85, 110]. Hailey and colleagues (2000) examined the impact of 20 rapid review products [43] and found that 14 had an influence on policy decision-making, four provided guidance, and two had no perceived impact. McGregor and Brophy (2005) evaluated the success of the conduct of 16 rapid reviews for a hospital rapid review service [60]. The results of all 16 products were directly implemented in the hospital, saving approximately $3 million per year. Hailey (2006) wrote a paper summarizing the impact of HTA in general, as well as related to rapid HTA. Overall, it was concluded that these reports can influence decision-making. Hailey (2009) conducted a survey of HTA organizations to examine the use of rapid reviews for decision-making [42]. Fifteen rapid review products were included; all influenced a decision, including using the rapid review for reference material (67 %) and directly using the rapid review’s conclusions for the decision (53 %). Zechmeister (2012) examined the impact of 58 rapid assessments and observed that 56 of these products were directly used for reimbursement decisions and two were used for disinvestment decisions [85]. Finally, Batten (2012) wrote an editorial discussing how rapid reviews can be used by school nurses [110].

## Discussion

Our results suggest that the conduct of rapid reviews is recondite across the literature. Through our study, 50 different rapid review approaches were identified and only 16 occurred more than once. Furthermore, many different terms were used to describe a rapid review, making the identification of these types of knowledge synthesis products difficult.

Using a framework of rapid review methods, we observed numerous strategies employed to conduct reviews in a streamlined manner. These included not using a protocol, limiting the literature search, limiting inclusion criteria, only having one person screen the literature search results, not conducting quality appraisal, and not conducting a meta-analysis. In general, combining multiple shortcuts led to a timelier conduct of the review.

Only four of the included studies compared the results of rapid reviews to systematic reviews. Three of these found that the results for both knowledge synthesis products were in agreement. However, the results of these studies should be interpreted with caution because a very small sample of reviews were included (ranging from 1 to 8) and none of these were prospectively conducted. The latter is of particular importance, since it is unclear whether the authors of the full systematic reviews used the rapid review as a starting point to identify articles for inclusion (or vice versa). Interestingly, none of the included studies compared the results across rapid reviews on the same topic. Such a study may provide further clarity into the impact of streamlining different steps on the risk of bias and comprehensiveness of the review.

Seven papers provided recommendations on making rapid reviews more efficient. Consistent guidance included using an integrated knowledge translation approach, limiting the scope of the question and literature search, and not conducting a meta-analysis. Furthermore, six papers examined the impact of rapid reviews on decision-making and all found that they were valuable products. These results suggest that decision-makers are currently using rapid reviews to inform their decision-making processes. Further supporting this observation was the recent Canadian Agency for Drugs and Technologies in Health *Rapid review summit* [116], for which a large number of international decision-making organizations were in attendance.

Across the application papers, many of the methods were poorly reported suggesting that improvement in the reporting of rapid reviews is warranted. Thorough reporting of the methods is important because it is difficult to judge the bias of these reports without fully understanding what shortcuts were taken. As well, transparent reporting allows the reproducibility of research. It is important to note that 10 % of the included papers were reported in 5 pages or less, suggesting that perhaps there was insufficient room to report the methods fully.

Prior to establishing a quality of reporting guidelines for rapid reviews, a common terminology and definition is required [117]. Some of the team members are currently involved with research that is attempting to tackle this issue. At the bare minimum, one of the included papers provided a checklist to examine the reporting of rapid reviews [12], which can be used by producers of rapid reviews to ensure their reports are reported in a consistent manner.

We have also conducted other research on rapid reviews that builds on this scoping review [118]. Specifically, we conducted an international survey of 40 rapid review producers who identified several rapid review approaches, such as updating the literature search of previous reviews and limiting the search strategy by date of publication. Most of the rapid review products were conducted within 12 weeks. A modified Delphi approach was used to include input from 113 stakeholders (for example, researchers, policy-makers, industry, journal editors, and healthcare providers) to agree upon an attractive rapid review method that would be used in a future comparative study. The stakeholders ranked the following method as being the most feasible, timely, and having a low perceived risk of bias: literature search limited by date and language; study selection by one reviewer only; and data abstraction and quality appraisal conducted by one reviewer and verified by a second reviewer. We are currently in the process of seeking funding of a comparative study to test the accuracy of this rapid review approach versus the gold standard, systematic review.

A recent project on rapid reviews was commissioned by the Agency for Healthcare Research and Quality in the United States [119, 120]. The authors summarized evidence from 12 review articles of rapid reviews [120], as well as 35 different rapid reviews produced by 20 different organizations [119]. This information was obtained through literature searches and key informant interviews with 18 individuals who had experience of conducting rapid reviews. The authors are currently conducting interviews with policy-makers to obtain their perceptions on rapid reviews, including their utility and importance.

Our scoping review has some limitations. To make our review more feasible, we were only able to include a random sample of rapid reviews from websites of rapid review producers. Further adding to this issue is that many rapid reviews contain proprietary information and are not publicly available. As such, our results are only likely generalizable to rapid reviews that are publicly available. Furthermore, this scoping review was an enormous undertaking and our results are only up to date as of May 2013. However, we believe that our results provide important information on rapid reviews and ours is the most comprehensive scoping review that we are currently aware of.

## Conclusions

In conclusion, numerous rapid review approaches were identified and few were used consistently in the literature. Poor quality of reporting was observed. Further research on rapid reviews is warranted. In particular, the consequences of various methodological shortcuts should be investigated. This could be examined through a prospective study comparing the results of rapid reviews to those obtained through systematic reviews on the same topic. Team members are currently seeking funding to conduct such a study and it is hoped that our results will provide pertinent information on the utility and risk of bias of rapid reviews.

## Additional file

Additional file 1:
**Includes five appendices with supplementary data.** (PDF 603 kb)

## Abbreviations

HTA: Health technology assessment
NR: Not reported
ROB: Risk of bias
SR: Systematic review
