# Supplementary material for: A scoping review of rapid review methods
Source: BMC Med. 2015 Sep 16;13:224. doi: 10.1186/s12916-015-0465-6 (PMC4574114; doi:10.1186/s12916-015-0465-6)
Supplement: Additional file 1: — Includes five appendices with supplementary data. (PDF 603 kb) [file 12916_2015_465_MOESM1_ESM.pdf]

Table of Contents

Appendix 1: MEDLINE Literature Search 2
Appendix 2: Framework of Rapid Review Methods 4
Appendix 3: Study Characteristics 6
Appendix 4: Terminology, definition, and rapid review citations 17
Appendix 5: Rapid review methods details 27

Appendix 1: MEDLINE Literature Search

Search Strategy:

----

1 (abbreviated adj review?).tw.

2 (abbreviated adj synthes?s).tw.

3 (accelerated adj2 review?).tw.

4 (accelerated adj2 synthes?s).tw.

5 (brief adj synthes?s).tw.

6 (expedited adj2 review?).tw.

7 (expedited adj2 synthes?s).tw.

8 (meta adj method$).tw.

9 (meta adj evaluat$).tw.

10 (rapid adj2 review?).tw.

11 (rapid adj2 assess$).tw.

12 "rapid health technology assess$".tw.

13 (rapid adj HTA?).tw.

14 (rapid adj approach$).tw.

15 (rapid adj search$).tw.

16 (rapid adj2 synthes?s).tw. not (ch or cs).fs.

17 (realis$ adj approach$).tw.

18 (realis$ adj evaluat$).tw.

19 (realis$ adj synthes?s).tw.

20 (speed$ adj2 review?).tw.

21 (streamline$ adj2 review?).tw.

22 (streamline$ adj2 synthes?s).tw.

23 (stream-line$ adj2 review?).tw.

24 (stream-line$ adj2 synthes?s).tw.

25 (fast$ adj2 review?).tw.

26 (fast$ adj2 synthes?s).tw.

27 or/1-26

28 exp Animals/ not (exp Animals/ and Humans/)

29 27 not 28

30 29 and ("2008$" or "2009$" or "2010$" or "2011$" or "2012$" or "2013$").ed.

Appendix 2: Framework of Rapid Review Methods

Rapid Review Steps			Coding Scheme
General	**Duration of Review**	> 6 months<br>≤ 6 months<br>Not reported	**SR method:** > 6 months<br>**Streamlined:** ≤ 6 months
	**Published protocol**	Yes<br>No<br>Not reported	**SR method:** protocol mentioned<br>**Streamlined:** protocol not mentioned
Literature Search	**Databases Searched**	Searched more than one database<br>Searched one database only<br>Used a previous review(s) as starting point<br>Not reported	**SR method:** at minimum, searched more than one database and searched grey literature<br>**Partially streamlined:** either **s**earched one database only / Used a previous review(s) as starting point and searched grey literature<br>**Fully streamlined:** database search not reported, grey lit search not reported, no grey literature search
	**Grey literature**	Searched grey literature<br>No grey literature search<br>Not reported	
Limits applied	**Date**	No limit<br>Limited by date<br>Not reported	**SR method:** no limit/ not reported<br>**Partially streamlined:** either date or language limited<br>**Fully streamlined:** both date and language limited
	**Language**	No limit<br>Limited by language<br>Not reported	
Screening	**Title & abstracts**	≥ 2 independent reviewers<br>One reviewer and one verifier<br>One reviewer only<br>Done but unclear number of reviewers<br>Not done<br>Not reported	**SR method**: both L1 and L2 screening had two independent reviewers<br>**Partially streamlined:** either L1 and L2 had one reviewer and one verifier<br>**Fully streamlined:** not done/ not reported, done but unclear
	**Full-texts**	≥ 2 independent reviewers<br>One reviewer and one verifier<br>One reviewer only<br>Done but unclear number of reviewers<br>Not done<br>Not reported	
Data Abstraction & Quality Appraisal	**Data abstraction**	≥ 2 independent reviewers<br>One reviewer and one verifier<br>One reviewer only<br>Done but unclear number of reviewers<br>Not done<br>Not reported	**SR method**: two independent reviewers<br>**Partially streamlined:** one reviewer and one verifier<br>**Fully streamlined:** one reviewer only, done but unclear number of reviewers, not done, not reported
	**Quality appraisal**	≥ 2 independent reviewers<br>One reviewer and one verifier<br>One reviewer only<br>Done but unclear number of reviewers<br>Not done<br>Not reported	**SR method:** two independent reviewers<br>**Partially streamlined:** one reviewer and one verifier/ one reviewer only/ done but unclear number of reviewers<br>**Fully streamlined:** not done/ not reported

**Data Synthesis**	**Data synthesis**	Meta-analysis and narrative summary<br>Meta-analysis/pooling only<br>Clear reasons for not doing meta-analysis<br>Narrative/descriptive summary only<br>Not reported	**MA/clear reasons:** meta-analysis and narrative summary/ clear reasons for not doing meta-analysis<br>**Descriptive:** narrative/descriptive summary only/ unclear or limited data synthesis methods/ not reported

Appendix 3: Study Characteristics

Author, Year	Type of Source (Report or Journal)	Country	Name of synthesis method	Type of review (application only)	Article Type	Duration of Review	Full Methods Reported	Published protocol	Review question
Abrami, 2010	Journal	Canada	Brief Review	NA	Development	6 months	Yes	Protocol not reported	Research question clearly reported
Adi, 2004	Report	UK	Rapid Systematic Reviews	Interventions	Application	3-6 months	Yes	Protocol not reported	Research question clearly reported
Armitage, 2009	Journal	UK	Rapid Structured Literature Review	Interventions	Application	NR	Yes	Protocol not reported	Research question clearly reported
Atree, 2008	Journal	UK	Rapid Review	Interventions	Application	12 months	Yes	Protocol not reported	Research question clearly reported
Australian Safety and Efficacy Register of New Interventional Procedures – Surgical, 2009	Report	Australia	Brief Review	Interventions	Application	NR	Yes	Protocol not reported	Research question clearly reported
Bambra, 2010	Journal	UK	Rapid (non-systematic) Narrative Literature Review	Interventions	Application	6 months	Yes	Protocol not reported	Research question clearly reported
Barnighausen, 2012	Journal	South Africa,	Rapid Review	Interventions	Application	NR	Yes	Protocol not reported	Research question

		USA, France, UK							clearly reported
Batten, 2012	Journal	US	Rapid Evidence Assessment	NA	Development	6 months	Yes	Protocol not reported	Research question clearly reported
Beck, 2012	Report	UK	Rapid Systematic Review of Evidence	Interventions	Application	NR	Yes	Protocol not reported	Research question clearly reported
Best, 1997	Journal	UK	Rapid and Responsive Health Technology Assessment	NA	Development	2 months	Yes	Protocol not reported	Research question unclear/in ferred
Best, 2012	Journal	Canada	Rapid Realist Review	Frequency	Application	6 months	Yes	Protocol not reported	Research question clearly reported
Birmingham and Black County Strategic Health Authority, 2008	Report	UK	Rapid Review	Interventions	Application	NR	Yes	Protocol not reported	Research question clearly reported
Blank, 2012	Journal	UK	Rapid Evidence Assessment	Frequency	Application	NR	Yes	Protocol not reported	Research question clearly reported
Boycott, 2012	Journal	UK	Rapid Evidence Assessment	Interventions	Application	NR	Yes	Protocol not reported	Research question clearly reported
Brearley, 2011	Journal	UK	Rapid Review and Synthesis of the Literature	Experience	Application	NR	Yes	Protocol not reported	Research question clearly reported
Brown, 2008	Report	Canada	Health Technology Assessment	Interventions	Application	NR	Yes	Protocol not reported	Research question clearly

			Rapid Review						reported
Brunton, 2013	Report	UK	Systematic Rapid Evidence Review (SREA)	Interventions	Application	9 weeks	Yes	Protocol not reported	Research question clearly reported
Bryant, 2006	Journal	UK	Rapid Literature Review	Frequency	Application	NR	Yes	Protocol not reported	Research question clearly reported
Bullock, 2010	Journal	USA	Expedited Systematic Review	Interventions	Application	NR	Yes	Protocol not reported	Research question clearly reported
Bungay, 2013	Journal	UK	Rapid Review	Interventions	Application	NR	Yes	Protocol not reported	Research question clearly reported
Burls, 2002	Journal	UK	Rapid Review	Interventions	Application	4-6 weeks	Yes	Protocol not reported	Research question clearly reported
Butler, 2004	Report	UK	Rapid Evidence Assessment	Interventions	Application	3 months	Yes	Protocol not reported	Research question clearly reported
Butt, 2010	Journal	UK	Rapid Systematic Review	Association	Application	NR	Yes	Protocol not reported	Research question clearly reported
Caird, 2012	Report	UK	Systematic Rapid Evidence Review (SREA)	Diagnosis	Application	NR	Yes	Protocol not reported	Research question clearly reported
Cameron, 2007 [CR: Watt 2008]	Report	Australia	Rapid Review	NA	Comparison	< 1 month	Yes	Protocol not reported	Research question clearly

									reported
Carr, 2011	Report	UK, New Zealand, India, Ireland	Rapid Evidence Assessment project	Interventions	Application	NR	Yes	Yes, published protocol	Research question clearly reported
Casadesus, 2009	Journal	Cuba	Rapid Review	Interventions	Application	NR	No	Protocol not reported	Research question unclear/inferred
Clark, 2003	Report	UK	Health Technology Assessment/Rapid Review	Interventions	Application	3 months	Yes	Protocol not reported	Research question clearly reported
Coomber, 2004	Report	UK	Rapid Interim Review	Interventions	Application	NR	Yes	Protocol not reported	Research question clearly reported
Corabian, 2002	Conference abstract	NR	Technotes	NA	Comparison	NR	No	Protocol not reported	NR
Cragg, 2008	Report	UK	Rapid Evidence Review and Appraisal	Association	Application	4 months	Yes	Protocol not reported	Research question clearly reported
Cummins, 2001	Report	UK	Rapid Review	Interventions	Application	NR	Yes	Protocol not reported	Research question clearly reported
Curson, 2010	Journal	UK	Rapid Review	Frequency	Application	3 months	No	NA	NA
De Alwis KLNSK, 2010	Journal	Australia	Rapid Evidence-based Literature Review	Interventions	Application	NR	No	NA	NA
De Laet, 2008	Report	Belgium	Rapid Assessment	Interventions	Application	NR	Yes	Protocol not reported	Research question clearly reported
Dixon-Woods,	Journal	UK	Literature	Frequency	Application	NR	Yes	Protocol not	Research

2012			Review					reported	question clearly reported
Doran, 2013	Report	Australia	Rapid Review (Evidence Check Review)	Interventions	Application	NR	Yes	Protocol not reported	Research question clearly reported
Fitzpatrick-Lewis, 2011	Journal	Canada	Rapid Systematic Review	Interventions	Application	NR	Yes	No protocol published	Research question clearly reported
Foerster, 2007	Report	Canada	Health Technology Assessment/Rapid Review	Interventions	Application	NR	Yes	Protocol not reported	Research question clearly reported
Government Social Research 2007	Report	UK	Rapid Evidence Assessments	NA	Development	8-12 weeks	Yes	Protocol not reported	Research question clearly reported
Geddes, 2011	Journal	UK	Rapid Review	Interventions	Application	NR	Yes	Protocol not reported	Research question clearly reported
Hailey, 2000	Journal	Australia	Rapid Health Technology Assessment (locally known as "Technotes")	NA	Evaluation	NR	Yes	Protocol not reported	Research question clearly reported
Hailey, 2006	Journal	Canada, Australia	Health Technology Assessment	NA	Evaluation	NR	Yes	NA	NA
Hailey, 2009	Journal	Canada	Rapid Health Technology Assessments	NA	Evaluation	1-6 months	Yes	NA	NA
Hildon, 2012	Journal	UK	Rapid Review	Frequency	Application	NR	Yes	Protocol not reported	Research question clearly

									reported
Hulstaert, 2009	Report	Belgium	Rapid Assessment	Interventions	Application	NR	Yes	Protocol not reported	Research question clearly reported
Jahangirian, 2011	Journal	UK	Rapid Review	NA	Development	NR	Yes	Protocol not reported	Research question clearly reported
Joliffe, 2007	Report	UK	Rapid Evidence Assessment	Interventions	Application	NR	Yes	Protocol not reported	Research question clearly reported
Kelly, 2011	Journal	Australia	Rapid Review	Interventions	Application	NR	Yes	Protocol not reported	Research question clearly reported
Khangura, 2012	Journal	Canada	Evidence Summaries	NA	Development	16 months	Yes	Protocol not reported	NA
Konnyu, 2012	Journal	Canada	Rapid Review	Interventions	Application	1 month	Yes	Protocol not reported	Research question clearly reported
Legrand, 2008	Report	Belgium	Rapid Assessment	Interventions	Application	NR	Yes	Protocol not reported	Research question clearly reported
Lewis, 2001	Journal	UK	Rapid and Systematic Review	Interventions	Application	NR	Yes	Protocol not reported	Research question clearly reported
Low, 2006	Report	Switzerland, UK	Rapid Review	Screening	Application	NR	Yes	Protocol not reported	Research question clearly reported
Maddern, [NR]	Report	Australia	Rapid Review/Rapid Systematic Review	Interventions	Application	NR	Yes	Protocol not reported	Research question clearly reported

Maddern, [NR]	Report	Australia	Rapid Systematic Review	Diagnosis	Application	NR	Yes	Protocol not reported	Research question clearly reported
Maddern, [NR]	Report	Australia	Rapid Systematic Review	Interventions	Application	NR	Yes	Protocol not reported	Research question clearly reported
Maddern, [NR]	Report	Australia	Rapid Systematic Review	Interventions	Application	NR	Yes	Protocol not reported	Research question clearly reported
Maddern, 2008	Report	Australia	Rapid Systematic Review	Interventions	Application	NR	Yes	Protocol not reported	Research question clearly reported
Mann, 2011	Journal	UK	Rapid Review	Diagnosis	Application	NR	Yes	Protocol not reported	Research question clearly reported
Marsh, 2008	Journal	UK	Rapid Evidence Assessment/Rapid Review	Interventions	Application	NR	Yes	Protocol not reported	Research question clearly reported
McGregor, 2005	Journal	Canada	Validation Study	NA	Evaluation	NR	Yes	NA	NA
Mcmurran, 2012	Journal	UK	Rapid Evidence Assessment	Interventions	Application	NR	Yes	Protocol not reported	Research question clearly reported
McRobbie, 2006	Report	UK, New Zealand	Rapid Review	Interventions	Application	NR	Yes	Protocol not reported	Research question clearly reported
Medical Advisory Secretariat, 2010	Report	Canada	Rapid Review	Interventions	Application	NR	Yes	Protocol not reported	Research question clearly reported
Middleton,	Report	Australia	Accelerated	Interventions	Application	NR	Yes	Protocol not	Research

2003			Systematic Review					reported	question clearly reported
Mitchell, 2011	Journal	USA	Rapid Health Technology Assessment (Evidence inventory report)	Frequency	Application	3-4 days	Yes	Protocol not reported	Research question clearly reported
Moran, 2011	Journal	UK	Rapid Review	Interventions	Application	NR	Yes	Protocol not reported	Research question clearly reported
Moyad, 2008	Journal	USA	Rapid Review	Interventions	Application	NR	No	Protocol not reported	Research question clearly reported
Moyad, 2011	Journal	USA	Rapid Review / quick review	Interventions	Application	NR	No	NA	NA
Murphy, 2010	Report	Canada	Rapid Review	Interventions	Application	NR	Yes	Protocol not reported	Research question clearly reported
Nasser, 2011	Journal	UK	Rapid Assessment	Interventions	Application	NR	Yes	Protocol not reported	Research question clearly reported
Ndegwa, 2010	Report	Canada	Rapid Review	Interventions	Application	NR	Yes	Protocol not reported	Research question clearly reported
O' Meara, 2001	Journal	UK, Netherlands	HTA/Rapid and Systematic Review	Interventions	Application	11 months	Yes	Protocol not reported	Research question clearly reported
Obyn, 2008	Report	Belgium	Rapid Assessment	Interventions	Application	NR	Yes	Protocol not reported	Research question clearly

									reported
Parker, 2006	Report	UK	Rapid Experience Review	Interventions	Application	NR	No	NA	NA
Phillipson, 2012	Report	Australia	Rapid Review (evidence check review)	Interventions	Application	NR	Yes	Protocol not reported	Research question clearly reported
Rissel, 2012	Report	Australia	Rapid Review/ Comprehensive rapid literature review/ evidence check review	Association	Application	NR	Yes	Protocol not reported	Research question clearly reported
Saborido, 2010	Journal	UK	Overview of Rapid Reviews	Interventions	Application	4 months	Yes	Protocol not reported	Research question clearly reported
Schnell-Inderst, 2011	Journal	Germany	Rapid assessment	Screening	Application	NR	Yes	Protocol not reported	Research question clearly reported
Singh, 2005	Report	UK	Rapid Review	Interventions	Application	NR	Yes	Protocol not reported	Research question clearly reported
Singh, 2006	Report	UK	Rapid Review	Frequency	Application	NR	Yes	Protocol not reported	Research question clearly reported
Singh, 2006	Report	UK	Rapid Review	Frequency	Application	3 weeks	Yes	Protocol not reported	Research question clearly reported
Smith, 2013	Journal	UK	Rapid Structured review	Experience	Application	NR	Yes	Protocol not reported	Research question clearly

									reported
Stordeur, 2009	Report	Belgium	Rapid Assessment	Interventions	Application	NR	Yes	Protocol not reported	Research question clearly reported
Sutton, 2011	Journal	UK	Rapid Review	Interventions	Application	NR	Yes	Protocol not reported	Research question clearly reported
Thavaneswaran , 2009	Report	Australia	Rapid Systematic Review	Interventions	Application	NR	Yes	Protocol not reported	Research question clearly reported
Thigpen, 2012	Journal	USA	Rapid Synthesis and Translation Process	NA	Development	10-12 months	Yes	Protocol not reported	Research question clearly reported
Thomas, 2013	Journal	UK	Rapid Evidence Assessment	NA	Development	NR	No	Protocol not reported	NA
Tonmukayakul, 2012	Journal	Thailand	Rapid Review	Interventions	Application	NR	Yes	Protocol not reported	Research question clearly reported
Tripney, 2011	Report	UK	Rapid Review	Interventions	Application	6 months	Yes	Yes, published protocol	Research question clearly reported
Tsakonas, 2008	Report	Canada	HTA Rapid Review	Diagnosis	Application	NR	Yes	Protocol not reported	Research question clearly reported
Van Brabandt, 2009	Report	Belgium	Rapid Health Technology Assessment	Interventions	Application	NR	Yes	Protocol not reported	Research question clearly reported
Van Branadt, 2008	Report	Belgium	Rapid Health Technology Assessment	Interventions	Application	NR	Yes	Protocol not reported	Research question clearly

									reported
Van de Velde, 2011	Journal	Belgium	Rapid Review	NA	Comparison	1 month	Yes	Protocol not reported	Research question not Reported
van Swieten, 2008	Journal	Netherlands	Rapid Review	Association	Application	NR	No	NA	NA
Vlayen, 2006	Report	Belgium, Netherlands	Rapid Assessment	Interventions	Application	NR	Yes	Protocol not reported	Research question clearly reported
WHO, 2006	Report	NR	Rapid Advice Guidelines	Interventions	Application	6 months	Yes	Protocol not reported	Research question clearly reported
York, 2011	Journal	US	Rapid Evidence Assessment of the Literature (REAL)	Frequency	Application	NR	Yes	Protocol not reported	Research question clearly reported
Zechmeister, 2012	Journal	Austria	Overview of HTAs	NA	Evaluation	NR	Yes	NA	NA

Appendix 4: Terminology, definition, and rapid review citations

Author, Year	Name of Synthesis Method	Definition of Rapid Review	Rapid Review Methods Cited
Adi, 2004	Rapid Systematic Reviews	Rapid systematic reviews about the effectiveness of healthcare interventions and technologies are produced in response to requests from West Midlands Health Authorities or the HTA programme. Reviews usually take 3-6 months and aim to give a timely and accurate analysis of the quality, strength and direction of the available evidence, generating an economic analysis (where possible a cost utility analysis) of the intervention.	West Midlands HTA Collaboration, The Trent Working Group on Acute Purchasing, The Wessex Institute for Health Research and Development, and The York Centre for Reviews and Dissemination
Armitage, 2009	Rapid Structured Literature Review	Rapid structured Literature Review has three major stages: conceptualization (the need and problem definition); operational aspects (conducting the literature search); structuring and reporting the RSLR (reporting).	NR
Attree, 2011	Rapid Review	No definition	NR
Australian Safety and Efficacy Register of New Interventional Procedures – Surgical, 2009	Brief Review	Brief report, while broad in some aspects of systematic review methodology, should not be considered to be a comprehensive systematic review.	NR
Bambra, 2010	Rapid(non-systematic) Narrative Literature Review	Drawing on the principles of systematic review methodology to identify relevant and accessible evidence within a limited time frame to feed into policy-making	NR
Barnighausen, 2012	Rapid Review	Rapid reviews are distinct from systematic reviews in that they typically apply stricter inclusion criteria and search fewer databases and sources than systematic reviews	Ganann R, Ciliska D, Thomas H. Expediting systematic reviews: methods and implications of rapid reviews. Implement Sci. 2010; 5: 56.<br><br>Watt A, Cameron A, Sturm L, et al. Rapid reviews versus full systematic reviews: an inventory of current methods and practice in health technology assessment. Int J

			Technol Assess Healthcare. 2008; 24: 133-139.
Batten, 2012	Rapid Evidence Assessment	Rapid evidence assessments are briefer than systematic reviews yet still rely on a focused research question, quality appraisal, and synthesis of research.	Civil Service (n.d.). What is a rapid evidence assessment? Retrieved September 18, 2012, from http://www.civilservice.gov.uk/networks/gsr/resources-and-guidance/rapid-evidence-assessment/what-is.
Beck, 2012	Rapid Systematic Review of evidence	In order to produce a robust but also timely appraisal of the evidence, a rapid review methodology was adopted, with the aim of creating a baseline resource which could be used in future for further work.	NR
Best, 1997	Rapid and responsive health technology assessment	No definition	Stevens A, Colin-Jones D, Gabbay J. 'Quick and clean': authoritative health technology assessment for local health care contracting. Health Trends. 1995; 27(2):37-42.
Best, 2012	Rapid Realist Review	A rapid review of a restricted literature and should therefore be seen as preliminary	Greenhalgh T, Robert G, Macfarlane F. et al. Diffusion of innovations in service organisations: Systematic review and recommendations. The Millbank Quarterly. 2004; 82: 581-629.
Birmingham, 2008	Rapid Review	No definition	NR
Blank, 2012	Rapid Evidence Assessment	Rapid evidence assessment (REA) provides a 'balanced assessment of what is already known about a policy or practice issue, by using systematic review methods to search and critically appraise existing research'.	Rapid Evidence Assessment (2011). Retrieved from http://www.civilservice.gov.uk/networks/gsr/resources-and-guidance/rapid-evidence-assessment (on 5 April 2011).
Boycott, 2012	Rapid Evidence Assessment	Rapid evidence assessment provides an overview of existing research on a specific research topic, as well as a simple extraction and synthesis of the relevant data	Civil Service (n.d.). What is a rapid evidence assessment? 2011: http://www.civilservice.gov.uk/networks/gsr/resources-and-guidance/rapid-evidence-assessment/what-is
Brearley, 2011	Rapid Review	No definition	NR
Brown, 2008	Health Technology Assessment Rapid Review	No definition	NR
Brunton, 2013	Systematic	A rapid evidence assessment differs from more traditional	Ganann R, Ciliska D, Thomas H.

	Rapid Evidence Review (SREA)	systematic reviews in that it is conducted within a shorter timeframe, often on focused issues (Gough et al. 2012). However, these differences in methodology used to adapt to the tight timelines must be carefully described and their impact on the findings considered, in order for the research to remain transparent and accountable (Ganann et al. 2010).	Expediting systematic reviews: methods and implications of rapid reviews. Implementation Science. 2010; 5: 56. www.implementationscience.com/content/5/1/56. (Accessed 24 Oct 2012).
Bryant, 2006	Rapid Literature Review	No definition	NR
Bullock, 2010	expedited systematic Review	No definition	NR
Bungay, 2013	Rapid Review	Rapid reviews are literature reviews that are conducted systematically within a limited time frame	
Burls, 2002	Rapid Review	Rapid reviews are completed in a limited time to inform the appraisal and guideline development processes. The review brings together evidence on key aspects of the use of the technology concerned.	Burls A, Cummins C, Jordan R. et al. West Midlands Development and Evaluation Service (DES) Handbook. Birmingham: University of Birmingham; 1998.
Butler, 2004	Rapid Evidence Assessment	A systematic assessment of the best available evidence that follows the principles of a systematic review, but concessions are made in order to complete reviews in a shorter time scale. Grey literature is not included.	NR
Butt, 2010	Rapid Systematic Review	No definition	
Caird, 2012	Systematic Rapid Evidence Review (SREA)	A systematic rapid evidence assessment (SREA) represents the only way in which a broad policy question may be answered within a tight timescale.	NR
Cameron, 2007	Rapid Review	Any HTA report or systematic review that has taken between 1-6 months to produce which contains the elements of a comprehensive literature search. The concept of 'rapid review' is increasing, driven primarily by this need to engage with clinicians, consumers and policy makers in a timely manner in order to provide evidence-based recommendations pertaining to healthcare activities and decisions.	NR
Carr, 2011	Rapid Evidence Assessment	No definition	NR

	project		
Clark, 2003	Health Technology Assessment/Rapid Review	No definition	NR
Coomber, 2004	Rapid Interim Review	No definition	NR
Cragg, 2008	Rapid Evidence Review and Appraisal	No definition	NR
Cummins, 2001	Rapid Review	No definition	NR
De Laet, 2008	Rapid Assessment	No definition	NR
Dixon-Woods, 2012	Literature Review	No definition	NR
Doran, 2013	Rapid Review (evidence check Review)	It is not necessarily a comprehensive review of all literature relating to the topic area.	NR
Fitzpatrick-Lewis, 2011	Rapid Systematic Review	No definition	NR
Foerster, 2007	Health Technology Assessment/Rapid Review	The report is based on a limited literature search and is not comprehensive, systematic reviews. The intent is to provide a list of sources and a summary of the best evidence on the topic that CADTH could identify using all reasonable efforts within the time allowed.	NR
Geddes, 2011	Rapid Review	Rapid reviews are literature reviews that use methods to accelerate or streamline traditional systematic review processes. Target audiences for rapid reviews include government policymakers, healthcare institutions, health professionals, and patient associations to inform health system planning and policy development	Ganann R, Ciliska D, Thomas H. Expediting systematic reviews: methods and implications of rapid reviews. Implement Sci. 2010; 5:56.
Government Social Research 2007	Rapid Review	No definition	NR
Hildon, 2012	Rapid Review	No definition	NR
Hulstaert, 2009	Rapid Assessment	No definition	NR
Jahangirian,	Rapid Review	No definition	NR

2011			
Joliffe, 2007	Rapid Evidence Assessment	The main difference between a systematic review and a REA is the restriction of the time period available to search for eligible studies	NR
Kelly, 2011	Rapid Review	Rapid reviews are a new and contentious approach to knowledge transfer and exchange between researchers and policy makers. They are typically conducted in months rather than years and the focus is determined by policy-makers rather than researchers. The strength of a rapid review lies in its timeliness and responsiveness to the needs of policy makers and practitioners, but its limitations include the reliability and generalisability of the findings.	Gannan R, Cilska D, Thomas H. Expediting systematic reviews: methods and implications of rapid reviews. Implementation Science 2010; 5(56).<br><br>Abrami PC, Borokhovski E, Bernerd R, et al. Issues in conducting and disseminating brief reviews of evidence. Evidence and Policy. 2010; 6(3): 371-89.<br><br>Butler G, Deaton S, Hodgkinson J, et al. Quick but not dirty: rapidevidence assessments as a decision support tool in social policy. Government Social Research Unit, 2005. London; 2005.
Konnyu, 2012	Rapid Review	Rapid reviews have emerged as a streamlined approach to synthesizing evidence quickly, typically for the purpose of helping decision-makers in health care and health services settings respond in a timely manner to urgent and emerging needs	Ganann R, Ciliska D, Thomas H. Expediting systematic reviews: methods and implications of rapid reviews. Implement Sci. 2010; 5: 56.<br><br>Information services CADTH. Grey matters: A practical search tool for evidence-based medicine. Canadian Agency for Drugs and Technologies in Health. 2011. Available from: http://www.cadth.ca/resources/grey-matters.<br><br>Watt A, Cameron A, Sturm L. et al. Rapid versus full systematic reviews: Validity in clinical practice? ANZ J Surg. 2008; 78(11): 1037-1040. SUPPORT Collaboration. SUPPORT: Supporting Policy Relevant Reviews and Trials. 2011. Available from www.support-collaboration.org.

Legrand, 2008	Rapid Assessment	No definition	NR
Lewis, 2001	Rapid and Systematic Review	Rapid reviews are completed in a limited time to inform the appraisal and guideline development processes. The review brings together evidence on key aspects of the use of the technology concerned.	NR
Low, 2006	Rapid Review	No definition	NR
Maddern, [NR]	Rapid Review/rapid Systematic Review	A rapid systematic review in which the methodology has been limited in one or more areas to shorten the timeline for its completion. This rapid review is a limited evidence-based assessment that is based on a simple systematic search of studies published in the peer reviewed literature. As a result, this rapid review may be used to inform certain questions on the specific review topic.	NR
Maddern, [NR]	Rapid Systematic Review	A rapid systematic review in which the methodology has been limited in one or more areas to shorten the timeline for its completion. This rapid review is a limited evidence-based assessment that is based on a simple systematic search of studies published in the peer reviewed literature. As a result, this rapid review may be used to inform certain questions on the specific review topic.	NR
Maddern, [NR]	Rapid Systematic Review	A rapid systematic review in which the methodology has been limited in one or more areas to shorten the timeline for its completion. This rapid review is a limited evidence-based assessment that is based on a simple systematic search of studies published in the peer reviewed literature. As a result, this rapid review may be used to inform certain questions on the specific review topic.	NR
Maddern, [NR]	Rapid Systematic Review	A rapid systematic review in which the methodology has been limited in one or more areas to shorten the timeline for its completion. This rapid review is a limited evidence-based assessment that is based on a simple systematic search of studies published in the peer reviewed literature. As a result, this rapid review may be used to inform certain questions on the specific review topic.	NR
Maddern, 2008	Rapid Systematic Review	A rapid systematic review in which the methodology has been limited in one or more areas to shorten the timeline for its completion. This rapid review is a limited evidence-based assessment that is based on a simple systematic search of studies published in the peer reviewed literature. As a result, this rapid	NR

		review may be used to inform certain questions on the specific review topic.	
Mann, 2011	Rapid Review	No definition	NR
Marsh, 2008	Rapid Evidence Assessment/Rapid Review	No definition	NR
Mcmurran, 2012	Rapid Evidence Assessment	A briefer version of a full systematic review, devised originally for informing policy decision-making quickly	Civil Service (2009) REA methods – Rapid Evidence Assessment. Retrieved June 13, 2011 from http://www.civilservice.gov.uk/my-civil-service/networks/professional/gsr/resources/rea-methodsrapid-evidence-assessment.aspx
McRobbie, 2006	Rapid Review	No definition	NR
Middleton, 2003	Accelerated Systematic Review	Accelerated systematic reviews are produced in response to a pressing need for a systematic summary and appraisal of the available literature for a new or emerging surgical procedure. They use the same methodology as full systematic reviews, but may restrict the types of studies considered (for example, by only including comparative studies and not case series) in order to produce the review in a shorter time period than a full systematic review.	NR
Mitchell, 2011	Rapid Health Technology Assessment (Evidence inventory report)	Expedited production of short-form evidence inventory reports. In an evidence inventory report the authors leave the 'patient' element of PICO unspecified and then assess how much evidence there is for the intervention in question, stratifying by patient group. The inventory process would work equally well assessing the quantity of evidence on different variations of a particular intervention for a particular patient group.	NR
Moran, 2011	Rapid Review	No definition	NR
Murphy, 2010	Rapid Review	No definition	NR
Nasser, 2011	Rapid Assessment	No definition	NR
Ndegwa, 2010	Rapid Review	No definition	NR
Obyn, 2008	Rapid Assessment	No definition	NR
O'Meara, 2001	HTA/Rapid and Systematic	Reviews in Health Technology Assessment are termed systematic when the account of the search appraisal and	NR

	Review	synthesis methods would, in theory, permit the replication of the review by others. Rapid reviews are completed in a limited time to inform the appraisal and guideline development processes managed by NICE.	
Phillips, 2012	Rapid Review (evidence check Review)	This evidence check review was produced using the evidence check methodology in response to specific questions from the commissioning agency. It is not necessarily a comprehensive review of all literature relating to the topic area.	NR
Rissel, 2012	Rapid Review/ Comprehensive rapid literature Review/ evidence check Review	This evidence check review was produced using the evidence check methodology in response to specific questions from the commissioning agency. It is not necessarily a comprehensive review of all literature relating to the topic area.	NR
Saborido, 2010	Overview of Rapid Reviews	No definition	NR
Schnell-Inderst, 2011	Rapid assessment	No definition	NR
Singh, 2005	Rapid Review	No definition	NR
Singh, 2006a	Rapid Review	No definition	NR
Singh, 2006b	Rapid Review	No definition	NR
Smith, 2013	Rapid structured Review	Rapid structured reviews are used to summarize and synthesis research findings within the constraints of a given timetable and resources and differ from systematic review in relation to the extensiveness of the literature search and methods used to undertake the analysis	Armitage A, Keeble-Ramsay D. The rapid structured literature review as a research strategy. Education Review. 2009; 6: 27-37.
Stordeur, 2009	Rapid Assessment	No definition	NR
Sutton, 2011	Rapid Review	Assessment of what is already known about a policy or practice issue, by using systematic review methods to search and critically appraise existing research. Rapid reviews, by their very nature, are constrained by time, and this impact on the completeness of searching undertaken and the depth of formal quality assessment. Nonetheless, this type of review can provide a general indication of the overall quality and direction of effect reported in the literature.	Grant MJ, Booth A. A typology of reviews: an analysis of 14 review types and associated methodologies. Health Information & Libraries Journal. 2009; 26: 91-108.
Thavaneswaran, 2009	Rapid systematic Review	A rapid systematic review in which the methodology has been limited in one or more areas to shorten the timeline for its completion. This rapid review is a limited evidence-based	NR

		assessment that is based on a simple systematic search of studies published in the peer reviewed literature. As a result, this rapid review may be used to inform certain questions on the specific review topic.	
The Medical Advisory Secretariat, 2010	Rapid Review	No definition	NR
Thigpen, 2012	Rapid synthesis and translation process	The Rapid Synthesis and Translation Program is collaborative and by using Lomas' exchange model of knowledge transfer bridges the gap between research and practice by keeping researchers and practitioners involved from the beginning. The RSTP borrows from other policy and science synthesis and translation literature much of which is related to knowledge transfer includes processes or components related to translation.	Ganann R, Ciliska D, Thomas H. Expediting systematic reviews: methods and implications of rapid reviews. Implementation Science. 2010; 5: 56.<br><br>Jack S, Tommyr L. Knowledge transfer and exchange: Disseminating Canadian child maltreatment surveillance findings to decision makers. Child Indicators Research. 2008; 1: 51-64.<br><br>Reardon R, Lavis J, Gibson J. From research to practice: A knowledge transfer planning guide. Toronto, ON: Institute for Work and Health.<br><br>Mallonee S, Fowler C, Istre GR. Bridging the gap between research and practice: A continuing challenge. Injury prevention. 2006; 12: 357-359. Robert Wood Johnson Foundation. (2009). The synthesis project. Retrieved from http://rwjf.org/pr/synthesis/about.jsp.
Tripney, 2011	Rapid Review	No definition	NR
Tsakonas, 2008	HTA rapid Review	HTIS responses are based on a limited literature search and are not comprehensive, systematic reviews. The intent is to provide a list of sources and a summary of the best evidence on the topic that CADTH could identify using all reasonable efforts within the time allowed. HTIS responses should be considered along with other types of information and health care considerations. The information included in this response is not intended to replace professional medical advice, nor should it be construed	NR

		as a recommendation for or against the use of a particular health technology.	
Van Branadt, 2008	Rapid Health Technology Assessment	No definition	NR
Van Branadt, 2009	Rapid Health Technology Assessment	No definition	NR
Vlayen, 2006	Rapid Assessment	No definition	NR
WHO, 2006	Rapid Advice Guidelines	No definition	NR
York, 2011	Rapid Evidence Assessment of the Literature (REAL)	The Rapid Evidence Assessment of the Literature approach differs from a conventional systematic literature review in that a REAL provides an evidence-based, systematic ‘‘snapshot’’ of the available literature.	NR

*NR, not reported

Appendix 5: Rapid review methods details

Author, Year	Definition	Duration of Review	General (published protocol, review questions)	Literature Search (i.e., databases, grey literature, reference scanning, contacting authors)	Inclusion Criteria (i.e., limited by data, language and study design)	Screening (i.e., title & abstract, full-text)	Data Abstraction and Quality Appraisal	Data Synthesis
Adi, 2004	Rapid systematic reviews about the effectiveness of healthcare interventions and technologies are produced in response to requests from West Midlands Health Authorities or the HTA programme. Reviews usually take 3-6 months and aim to give a timely and accurate analysis of the quality, strength and direction of the available evidence, generating an economic analysis (where possible a cost utility analysis) of the intervention.	3-6 months	Protocol not mentioned; Research question clearly reported	Searched more than one database; Searched published and grey literature; Search strategy clearly reported; Yes, references scanned; Yes, authors contacted	No Date limit; No Language limit; No limit on study design	L1 - two independent reviewers; L2 - Not reported	DA - One reviewer and one verifier; QA - One reviewer only	Narrative/descriptive summary only
Armitage, 2009	Rapid structured Literature Review has three major stages: conceptualization (the need and problem definition); operational aspects (conducting the	6 months	Protocol not mentioned; Research question clearly reported	Database search not reported; Searched published and grey literature; Search strategy clearly reported; Reference scanning	Data limit unclear/not reported; Language limit unclear/not reported; Study design	L1 - not reported; L2 - Not reported	DA - Done but unclear number of reviewers; QA - Done but unclear number of	Narrative/descriptive summary only

	literature search); structuring and reporting the RSLR (reporting)			not reported; Contacting authors not reported	unclear/not reported		reviewers	
Attree, 2011	No definition	12 months	Protocol not mentioned; Research question clearly reported	Searched more than one database; Searched published and grey literature; Search strategy not reported; Reference scanning not reported; Contacting authors not reported	Data limit unclear/not reported; Language limit unclear/not reported; No limit on study design	L1 - one reviewer and one verifier; L2 - One reviewer and one verifier	DA - Done but unclear number of reviewers; QA - Done but unclear number of reviewers	Narrative/descriptive summary only
Australian Safety and Efficacy Register of New Interventional Procedures – Surgical, 2009	Brief report, while broad in some aspects of systematic review methodology, should not be considered to be a comprehensive systematic review.	NR	Protocol not mentioned; Research question clearly reported	Searched more than one database; Searched published and grey literature; Search strategy clearly reported; Reference scanning not reported; Contacting authors not reported	Limited by date; Limited by language; Limited by study design	L1 - not reported; L2 - Not reported	DA - One reviewer only; QA - Not reported	Clear reasons for not doing meta-analysis
Bambra, 2010	Drawing on the principles of systematic review methodology to identify relevant and accessible evidence within a limited time frame to feed into policy-making	6 months	Protocol not mentioned; Research question clearly reported	Searched more than one database; Searched published sources only; Search strategy not reported; Reference scanning not reported; Contacting authors not reported	Limited by date; Language limit unclear/not reported; Limited by study design	L1 - not reported; L2 - Not reported	DA - Done but unclear number of reviewers; QA - Done but unclear number of reviewers	Narrative/descriptive summary only
Barnighausen, 2012	Rapid reviews are distinct from systematic reviews in that they typically apply stricter inclusion criteria and search fewer databases	NR	Protocol not mentioned; Research question clearly reported	Searched one database only; Searched published sources only; Search strategy clearly reported;	Limited by date; Language limit unclear/not reported; Limited by	L1 - not reported; L2 - Not reported	DA - One reviewer only; QA - Not reported	Narrative/descriptive summary only

	and sources than systematic reviews			Yes, references scanned; Yes, authors contacted	study design			
Batten, 2012	Rapid evidence assessments are briefer than systematic reviews yet still rely on a focused research question, quality appraisal, and synthesis of research.	6 months	Protocol not mentioned; Research question clearly reported	Database search not reported; Searched published sources only; Search strategy not reported; Reference scanning not reported; Contacting authors not reported	Data limit unclear/not reported; Language limit unclear/not reported; Study design unclear/not reported	L1 - not reported; L2 - Not reported	DA - Not reported; QA - Done but unclear number of reviewers	Unclear/limited data synthesis methods
Beck, 2012	In order to produce a robust but also timely appraisal of the evidence, a rapid review methodology was adopted, with the aim of creating a baseline resource which could be used in future for further work.	NR	Protocol not mentioned; Research question clearly reported	Searched more than one database; Searched published and grey literature; Search strategy clearly reported; Yes, references scanned; Contacting authors not reported	Limited by date; Limited by language; Limited by study design	L1 - Done but unclear number of reviewers; L2 - Done but unclear number of reviewers	DA - Done but unclear number of reviewers; QA - Not reported	Narrative/descriptive summary only
Best, 1997	No definition	2 months	Protocol not mentioned; Research question unclear/inferred	Searched more than one database; Searched published and grey literature; Search strategy clearly reported; Reference scanning not reported; Contacting authors not reported	Data limit unclear/not reported; Language limit unclear/not reported; No limit on study design	L1- not done; L2 - Not reported	DA - Not reported; QA - Done but unclear number of reviewers	Meta-analysis and narrative summary
Best, 2012	A rapid review of a restricted literature and should therefore be seen as preliminary	6 months	Protocol not mentioned; Research question clearly reported	Searched more than one database; Searched published and grey literature; Search strategy clearly reported; Yes, references	Limited by date; Language limit unclear/not reported; Study design unclear/not	L1 - two independent reviewers; L2 - Done but unclear number of reviewers	DA - Done but unclear number of reviewers; QA - Not reported	Narrative/descriptive summary only

				scanned; Contacting authors not reported	reported			
Birmingham and Black County Strategic Health Authority, 2008	No definition	NR	Protocol not mentioned; Research question clearly reported	Searched more than one database; Searched published and grey literature; Search strategy clearly reported; Yes, references scanned; Yes, authors contacted	Limited by date; No Language limit; Limited by study design	L1 - two independen t reviewers; L2 - Two independen t reviewers	DA - One reviewer and one verifier; QA - Done but unclear number of reviewers	Clear reasons for not doing meta-analysis
Blank, 2012	Rapid evidence assessment (REA) provides a 'balanced assessment of what is already known about a policy or practice issue, by using systematic review methods to search and critically appraise existing research'.	NR	Protocol not mentioned; Research question clearly reported	Searched more than one database; Searched published sources only; Search strategy clearly reported; Yes, references scanned; No authors contacted	Limited by date; Limited by language; Study design unclear/not reported	L1 - Done but unclear number of reviewers; L2 - Done but unclear number of reviewers	DA - One reviewer and one verifier; QA - One reviewer and one verifier	Clear reasons for not doing meta-analysis
Boycott, 2012	Rapid evidence assessment provides an overview of existing research on a specific research topic, as well as a simple extraction and synthesis of the relevant data	NR	Protocol not mentioned; Research question clearly reported	Searched more than one database; Searched published sources only; Search strategy clearly reported; Yes, references scanned; Yes, authors contacted	Limited by date; Limited by language; Limited by study design	L1 - one reviewer and one verifier; L2 - Not reported	DA - Done but unclear number of reviewers; QA - Done but unclear number of reviewers	Narrative/descriptive summary only
Brearley, 2011	No definition	NR	Protocol not mentioned; Research question clearly reported	Searched more than one database; Searched published sources only; Search strategy clearly reported; Reference scanning not reported;	Limited by date; Limited by language; Limited by study design	L1 - two independen t reviewers; L2 - One reviewer and one verifier	DA - One reviewer and one verifier; QA - One reviewer and one verifier	Narrative/descriptive summary only

				Contacting authors not reported				
Brown, 2008	No definition	NR	Protocol not mentioned; Research question clearly reported	Searched more than one database; Searched published and grey literature; Search strategy unclear; Yes, references scanned; Contacting authors not reported	Limited by date; Limited by language; No limit on study design	L1 - two independen t reviewers; L2 - Not reported	DA - Done but unclear number of reviewers; QA - Done but unclear number of reviewers	Narrative/d escriptive summary only
Brunton, 2013	A rapid evidence assessment differs from more traditional systematic reviews in that it is conducted within a shorter timeframe, often on focused issues (Gough et al. 2012). However, these differences in methodology used to adapt to the tight timelines must be carefully described and their impact on the findings considered, in order for the research to remain transparent and accountable (Ganann et al. 2010).	9 weeks	Protocol not mentioned; Research question clearly reported	Searched more than one database; Searched published and grey literature; Search strategy clearly reported; Yes, references scanned; Contacting authors not reported	Limited by date; Limited by language; Limited by study design	L1 - two independen t reviewers; L2 - Two independen t reviewers	DA - Two independen t reviewers; QA- Two independen t reviewers	Narrative/d escriptive summary only
Bryant, 2006	No definition	NR	Protocol not mentioned; Research question clearly reported	Searched one database only; Searched published and grey literature; Search strategy not reported; Reference scanning not reported; Contacting authors	Limited by date; Language limit unclear/not reported; Study design unclear/not reported	L1 - Done but unclear number of reviewers; L2 - Done but unclear number of reviewers	DA - Not reported; QA - Not reported	Narrative/d escriptive summary only

				not reported				
Bullock, 2010	No definition	NR	Protocol not mentioned; Research question clearly reported	Database search not reported; No grey literature mentioned; Search strategy unclear; Reference scanning not reported; Contacting authors not reported	Limited by date; Limited by language; Limited by study design	L1 - Done but unclear number of reviewers; L2 - Not reported	DA - Done but unclear number of reviewers; QA - One reviewer and one verifier	Narrative/descriptive summary only
Bungay, 2013	Rapid reviews are literature reviews that are conducted systematically within a limited time frame	NR	Protocol not mentioned; Research question clearly reported	Used a previous review(s) as starting point; Searched published and grey literature; Search strategy clearly reported; Reference scanning not reported; Contacting authors not reported	Limited by date; Limited by language; No limit on study design	L1 - Done but unclear number of reviewers; L2 - Done but unclear number of reviewers	DA - Done but unclear number of reviewers; QA - One reviewer only	Narrative/descriptive summary only
Burls, 2002	Rapid reviews are completed in a limited time to inform the appraisal and guideline development processes. The review brings together evidence on key aspects of the use of the technology concerned.	4-6 weeks	Protocol not mentioned; Research question clearly reported	Searched more than one database; Searched published and grey literature; Search strategy clearly reported; Yes, references scanned; Contacting authors not reported	Limited by date; No Language limit; Limited by study design	L1 - two independent reviewers; L2 - Two independent reviewers	DA - Two independent reviewers; QA- Two independent reviewers	Meta-analysis and narrative summary
Butler, 2004	A systematic assessment of the best available evidence that follows the principles of a systematic review, but concessions are made in order to complete reviews in a shorter time scale. Grey	3 months	Protocol not mentioned; Research question clearly reported	Searched more than one database; Searched published sources only; Search strategy clearly reported; Yes, references scanned; Contacting authors	Limited by date; Language limit unclear/not reported; Study design unclear/not reported	L1 - two independent reviewers; L2 - Two independent reviewers	DA - Done but unclear number of reviewers; QA - More than two reviewers	Narrative/descriptive summary only

	literature is not included.			not reported				
Butt, 2010	No definition	NR	Protocol not mentioned; Research question clearly reported	Searched more than one database; Searched published sources only; Search strategy clearly reported; Yes, references scanned; Yes, authors contacted	No Date limit; Limited by language; Limited by study design	L1 - one reviewer only; L2 - One reviewer only	DA - One reviewer only; QA - One reviewer and one verifier	Clear reasons for not doing meta-analysis
Caird, 2012	A systematic rapid evidence assessment (SREA) represents the only way in which a broad policy question may be answered within a tight timescale.	NR	Protocol not mentioned; Research question clearly reported	Searched more than one database; Searched published sources only; Search strategy clearly reported; Yes, references scanned; Yes, authors contacted	Limited by date; Limited by language; Limited by study design	L1 - Done but unclear number of reviewers; L2 - Not reported	DA - Done but unclear number of reviewers; QA- Two independen t reviewers	Narrative/d escriptive summary only
Cameron, 2007 [CR: Watt 2008b]	Any HTA report or systematic review that has taken between 1-6 months to produce which contains the elements of a comprehensive literature search. The concept of 'rapid review' is increasing, driven primarily by this need to engage with clinicians, consumers and policy makers in a timely manner in order to provide evidence-based recommendations pertaining to healthcare activities and decisions.	< 1 month	Protocol not mentioned; Research question clearly reported	Searched more than one database; Searched published and grey literature; Search strategy clearly reported; Yes, references scanned; Contacting authors not reported	Limited by date; Limited by language; Limited by study design	L1 - two independen t reviewers; L2 - Two independen t reviewers	DA - One reviewer and one verifier; QA - Done but unclear number of reviewers	Narrative/d escriptive summary only
Carr, 2011	No definition	NR	Yes,	Searched more	Limited by	L1 - two	DA - Two	Narrative/d

			protocol mentioned; Research question clearly reported	than one database; Searched published and grey literature; Search strategy clearly reported; Yes, references scanned; Contacting authors not reported	date; No Language limit; No limit on study design	independen t reviewers; L2 - Two independen t reviewers	independen t reviewers; QA- Two independen t reviewers	escriptive summary only
Clark, 2003	No definition	3 months	Protocol not mentioned; Research question clearly reported	Searched more than one database; Searched published and grey literature; Search strategy clearly reported; Yes, references scanned; Contacting authors not reported	No Date limit; No Language limit; No limit on study design	L1 - two independen t reviewers; L2 - Two independen t reviewers	DA - Two independen t reviewers; QA- Two independen t reviewers	Meta-analysis and narrative summary
Coomber, 2004	No definition	NR	Protocol not mentioned; Research question clearly reported	Used a previous review(s) as starting point; Searched published and grey literature; Search strategy clearly reported; Reference scanning not reported; Yes, authors contacted	Data limit unclear/not reported; Language limit unclear/not reported; No limit on study design	L1 - two independen t reviewers; L2 - Done but unclear number of reviewers	DA - Not reported; QA - Not done	Narrative/d escriptive summary only
Cragg, 2008	No definition	4 months	Protocol not mentioned; Research question clearly reported	Database search not reported; Searched published and grey literature; Search strategy not reported; Reference scanning not reported; Contacting authors not reported	Data limit unclear/not reported; Language limit unclear/not reported; Study design unclear/not reported	L1 - not reported; L2 - Not reported	DA - Done but unclear number of reviewers; QA - Done but unclear number of reviewers	Narrative/d escriptive summary only
Cummins,	No definition	NR	Protocol not	Searched more	Data limit	L1 - two	DA - Two	Narrative/d

2001			mentioned; Research question clearly reported	than one database; Searched published and grey literature; Search strategy clearly reported; Reference scanning not reported; Contacting authors not reported	unclear/not reported; No Language limit; Limited by study design	independent reviewers; L2 - Not reported	independent reviewers; QA - Done but unclear number of reviewers	escriptive summary only
De Laet, 2008	No definition	NR	Protocol not mentioned; Research question clearly reported	Searched more than one database; Searched published and grey literature; Search strategy clearly reported; Yes, references scanned; Contacting authors not reported	Limited by date; Language limit unclear/not reported; Limited by study design	L1 - Done but unclear number of reviewers; L2 - Done but unclear number of reviewers	DA - Not reported; QA - Not reported	Narrative/descriptive summary only
Dixon-Woods, 2012	No definition	NR	Protocol not mentioned; Research question clearly reported	Used a previous review(s) as starting point; No Grey literature mentioned; Search strategy not reported; Yes, references scanned; Contacting authors not reported	Data limit unclear/not reported; Language limit unclear/not reported; No limit on study design	L1 - not reported; L2 - Not reported	DA - Not reported; QA - Not done	Narrative/descriptive summary only
Doran, 2013	It is not necessarily a comprehensive review of all literature relating to the topic area.	NR	Protocol not mentioned; Research question clearly reported	Searched more than one database; Searched published and grey literature; Search strategy clearly reported; Yes, references scanned; Yes, authors contacted	Limited by date; Language limit unclear/not reported; Limited by study design	L1 - one reviewer only; L2 - One reviewer only	DA - Done but unclear number of reviewers; QA - Done but unclear number of reviewers	Narrative/descriptive summary only
Fitzpatrick-Lewis, 2011	No definition	NR	No protocol published;	Searched more than one database;	Limited by date; Limited	L1 - one reviewer	DA - One reviewer	Narrative/descriptive

			Research question clearly reported	Searched published and grey literature; Search strategy clearly reported; Yes, references scanned; Contacting authors not reported	by language; Limited by study design	and one verifier; L2 - Two independen t reviewers	and one verifier; QA- Two independen t reviewers	summary only
Foerster, 2007	The report is based on a limited literature search and is not comprehensive, systematic reviews. The intent is to provide a list of sources and a summary of the best evidence on the topic that CADTH could identify using all reasonable efforts within the time allowed.	NR	Protocol not mentioned; Research question clearly reported	Searched more than one database; Searched published and grey literature; Search strategy unclear; Reference scanning not reported; Contacting authors not reported	Limited by date; Limited by language; Study design unclear/not reported	L1 - Done but unclear number of reviewers; L2 - Done but unclear number of reviewers	DA - Done but unclear number of reviewers; QA - Not done	Narrative/d escriptive summary only
Geddes, 2011	No definition	NR	Protocol not mentioned; Research question clearly reported	Searched more than one database; Searched published and grey literature; Search strategy unclear; Reference scanning not reported; Contacting authors not reported	Limited by date; Language limit unclear/not reported; Limited by study design	L1 - not reported; L2 - Not reported	DA - Not reported; QA - Done but unclear number of reviewers	Narrative/d escriptive summary only
Hildon, 2012	No definition	NR	Protocol not mentioned; Research question clearly reported	Searched more than one database; Searched published and grey literature; Search strategy clearly reported; Reference scanning not reported;	Data limit unclear/not reported; Limited by language; Study design unclear/not reported	L1 - Done but unclear number of reviewers; L2 - Done but unclear number of reviewers	DA - Done but unclear number of reviewers; QA - Not done	Narrative/d escriptive summary only

				Contacting authors not reported			
Hulstaert, 2009	No definition	NR	Protocol not mentioned; Research question clearly reported	Searched more than one database; Searched published and grey literature; Search strategy clearly reported; Reference scanning not reported; Contacting authors not reported	Limited by date; Language limit unclear/not reported; Limited by study design	L1 - Done but unclear number of reviewers; L2 - Not reported	DA - Not done; QA - Not done	Narrative/descriptive summary only
Jahangirian, 2011	No definition	NR	Protocol not mentioned; Research question clearly reported	Searched more than one database; Searched published and grey literature; Search strategy unclear; Reference scanning not reported; Contacting authors not reported	Data limit unclear/not reported; Language limit unclear/not reported; Study design unclear/not reported	L1 - not reported; L2 - Not reported	DA - Done but unclear number of reviewers; QA - Not reported	Unclear/limited data synthesis methods
Joliffe, 2007	The main difference between a systematic review and a REA is the restriction of the time period available to search for eligible studies	NR	Protocol not mentioned; Research question clearly reported	Searched more than one database; Searched published and grey literature; Search strategy not reported; Reference scanning not reported; Yes, authors contacted	Data limit unclear/not reported; Limited by language; Study design unclear/not reported	L1 - Done but unclear number of reviewers; L2 - Done but unclear number of reviewers	DA - Not reported; QA - Not reported	Meta-analysis and narrative summary
Kelly, 2011	Rapid reviews are a new and contentious approach to knowledge transfer and exchange between researchers and policy makers. They are typically conducted in months rather than years and	NR	Protocol not mentioned; Research question clearly reported	Searched more than one database; Searched published and grey literature; Search strategy clearly reported; Yes, references scanned; Contacting authors	Limited by date; Language limit unclear/not reported; Study design unclear/not reported	L1 - one reviewer and one verifier; L2 - Not reported	DA - Done but unclear number of reviewers; QA - Done but unclear number of reviewers	Narrative/descriptive summary only

	the focus is determined by policy-makers rather than researchers. The strength of a rapid review lies in its timeliness and responsiveness to the needs of policy makers and practitioners, but its limitations include the reliability and generalisability of the findings.			not reported				
Konnyu, 2012	Rapid reviews have emerged as a streamlined approach to synthesizing evidence quickly, typically for the purpose of helping decision-makers in health care and health services settings respond in a timely manner to urgent and emerging needs	1 month	Protocol not mentioned; Research question clearly reported	Searched more than one database; Searched published and grey literature; Search strategy clearly reported; Yes, references scanned; Contacting authors not reported	Limited by date; Limited by language; Limited by study design	L1 - two independen t reviewers; L2 - Done but unclear number of reviewers	DA - One reviewer only; QA - One reviewer only	Clear reasons for not doing meta-analysis
Legrand, 2008	No definition	NR	Protocol not mentioned; Research question clearly reported	Searched more than one database; Searched published and grey literature; Search strategy clearly reported; Reference scanning not reported; Contacting authors not reported	Limited by date; Limited by language; Study design unclear/not reported	L1 - two independen t reviewers; L2 - Done but unclear number of reviewers	DA - Not reported; QA - Not reported	Narrative/d escriptive summary only
Lewis, 2001		NR	Protocol not mentioned; Research question clearly	Searched more than one database; Searched published and grey literature; Search strategy	No Date limit; Limited by language; Limited by study design	L1 - two independen t reviewers; L2 - Two independen	DA - One reviewer and one verifier; QA- Two	Meta-analysis and narrative summary

			reported	clearly reported; Yes, references scanned; Contacting authors not reported		t reviewers	independen t reviewers	
Low, 2006	No definition	NR	Protocol not mentioned; Research question clearly reported	Searched more than one database; Searched published and grey literature; Search strategy clearly reported; Yes, references scanned; Contacting authors not reported	Limited by date; No Language limit; No limit on study design	L1 - two independen t reviewers; L2 - Two independen t reviewers	DA - Two independen t reviewers; QA - Done but unclear number of reviewers	Narrative/d escriptive summary only
Maddern, [NR]	A rapid systematic review in which the methodology has been limited in one or more areas to shorten the timeline for its completion. This rapid review is a limited evidence-based assessment that is based on a simple systematic search of studies published in the peer reviewed literature. As a result, this rapid review may be used to inform certain questions on the specific review topic.	NR	Protocol not mentioned; Research question clearly reported	Searched more than one database; Searched published sources only; Search strategy clearly reported; No references scanned; No authors contacted	Limited by date; Limited by language; Limited by study design	L1 - one reviewer only; L2 - One reviewer only	DA - One reviewer and one verifier; QA - One reviewer and one verifier	Narrative/d escriptive summary only
Maddern, [NR]	A rapid systematic review in which the methodology has been limited in one or more areas to shorten the timeline for its	NR	Protocol not mentioned; Research question clearly reported	Searched more than one database; Searched published sources only; Search strategy clearly reported;	Limited by date; Limited by language; Limited by study design	L1 - one reviewer only; L2 - One reviewer only	DA - One reviewer and one verifier; QA - One reviewer	Narrative/d escriptive summary only

	completion. This rapid review is a limited evidence-based assessment that is based on a simple systematic search of studies published in the peer reviewed literature. As a result, this rapid review may be used to inform certain questions on the specific review topic.			No references scanned; No authors contacted			and one verifier	
Maddern, [NR]	A rapid systematic review in which the methodology has been limited in one or more areas to shorten the timeline for its completion. This rapid review is a limited evidence-based assessment that is based on a simple systematic search of studies published in the peer reviewed literature. As a result, this rapid review may be used to inform certain questions on the specific review topic.	NR	Protocol not mentioned; Research question clearly reported	Searched more than one database; Searched published sources only; Search strategy clearly reported; No references scanned; No authors contacted	Limited by date; Limited by language; Limited by study design	L1 - one reviewer only; L2 - One reviewer only	DA - One reviewer and one verifier; QA - One reviewer and one verifier	Narrative/descriptive summary only
Maddern, [NR]	A rapid systematic review in which the methodology has been limited in one or more areas to shorten the timeline for its completion. This rapid review is a limited	NR	Protocol not mentioned; Research question clearly reported	Searched more than one database; Searched published sources only; Search strategy clearly reported; No references scanned; No	No Date limit; Limited by language; Limited by study design	L1 - one reviewer only; L2 - One reviewer only	DA - One reviewer and one verifier; QA - One reviewer and one verifier	Narrative/descriptive summary only

	evidence-based assessment that is based on a simple systematic search of studies published in the peer reviewed literature. As a result, this rapid review may be used to inform certain questions on the specific review topic.			authors contacted				
Maddern, 2008	A rapid systematic review in which the methodology has been limited in one or more areas to shorten the timeline for its completion. This rapid review is a limited evidence-based assessment that is based on a simple systematic search of studies published in the peer reviewed literature. As a result, this rapid review may be used to inform certain questions on the specific review topic.	NR	Protocol not mentioned; Research question clearly reported	Searched more than one database; Searched published sources only; Search strategy clearly reported; No references scanned; No authors contacted	Limited by date; Limited by language; Limited by study design	L1 - one reviewer only; L2 - One reviewer only	DA - One reviewer and one verifier; QA - One reviewer and one verifier	Narrative/descriptive summary only
Mann, 2011	No definition	NR	Protocol not mentioned; Research question clearly reported	Searched more than one database; Searched published and grey literature; Search strategy clearly reported; Yes, references scanned; Contacting authors not reported	Limited by date; Limited by language; Limited by study design	L1 - Done but unclear number of reviewers; L2 - Done but unclear number of reviewers	DA - Done but unclear number of reviewers; QA - Done but unclear number of reviewers	Narrative/descriptive summary only

Marsh, 2008	No definition	NR	Protocol not mentioned; Research question clearly reported	Searched more than one database; Searched published sources only; Search strategy clearly reported; Reference scanning not reported; Contacting authors not reported	Limited by date; Limited by language; Limited by study design	L1 - two independen t reviewers; L2 - Two independen t reviewers	DA - Done but unclear number of reviewers; QA - Done but unclear number of reviewers	Meta-analysis and narrative summary
Mcmurran, 2012	A briefer version of a full systematic review, devised originally for informing policy decision-making quickly	NR	Protocol not mentioned; Research question clearly reported	Searched more than one database; Searched published and grey literature; Search strategy clearly reported; Reference scanning not reported; Yes, authors contacted	Limited by date; Limited by language; Limited by study design	L1 - one reviewer only; L2 - One reviewer only	DA - One reviewer only; QA - One reviewer only	Narrative/d escriptive summary only
McRobbie, 2006	No definition	NR	Protocol not mentioned; Research question clearly reported	Searched more than one database; Searched published and grey literature; Search strategy clearly reported; Reference scanning not reported; Contacting authors not reported	Limited by date; Limited by language; No limit on study design	L1 - one reviewer only; L2 - Two independen t reviewers	DA - Done but unclear number of reviewers; QA - Done but unclear number of reviewers	Meta-analysis and narrative summary
Middleton, 2003	Accelerated systematic reviews are produced in response to a pressing need for a systematic summary and appraisal of the available literature for a new or emerging surgical procedure. They use the same methodology as full systematic	NR	Protocol not mentioned; Research question clearly reported	Searched more than one database; Searched published sources only; Search strategy clearly reported; Reference scanning not reported; Contacting authors not reported	No Date limit; No Language limit; Limited by study design	L1 - not reported; L2 - Not reported	DA - One reviewer and one verifier; QA - Done but unclear number of reviewers	Clear reasons for not doing meta-analysis

	reviews, but may restrict the types of studies considered (for example, by only including comparative studies and not case series) in order to produce the review in a shorter time period than a full systematic review.							
Mitchell, 2011	Expedited production of short-form evidence inventory reports. In an evidence inventory report the authors leave the 'patient' element of PICO unspecified and then assess how much evidence there is for the intervention in question, stratifying by patient group. The inventory process would work equally well assessing the quantity of evidence on different variations of a particular intervention for a particular patient group.	3-4 days	Protocol not mentioned; Research question clearly reported	Searched more than one database; Searched published sources only; Search strategy unclear; Reference scanning not reported; Contacting authors not reported	Data limit unclear/not reported; Language limit unclear/not reported; Limited by study design	L1 - Done but unclear number of reviewers; L2 - Done but unclear number of reviewers	DA - Done but unclear number of reviewers; QA - Not reported	Narrative/descriptive summary only
Moran, 2011	No definition	NR	Protocol not mentioned; Research question clearly reported	Searched more than one database; Searched published and grey literature; Search strategy not reported; Reference scanning not reported; Contacting authors	Limited by date; Language limit unclear/not reported; Study design unclear/not reported	L1 - not reported; L2 - Not reported	DA - Not reported; QA - Not reported	Narrative/descriptive summary only

				not reported				
Murphy, 2010	No definition	NR	Protocol not mentioned; Research question clearly reported	Searched more than one database; Searched published and grey literature; Search strategy clearly reported; Yes, references scanned; Contacting authors not reported	Limited by date; Limited by language; Limited by study design	L1 - one reviewer only; L2 - One reviewer and one verifier	DA - Not reported; QA - Not reported	Narrative/descriptive summary only
Nasser, 2011	No definition	NR	Protocol not mentioned; Research question clearly reported	Searched more than one database; No Grey literature mentioned; Search strategy clearly reported; Reference scanning not reported; Contacting authors not reported	Limited by date; Language limit unclear/not reported; No limit on study design	L1 - not reported; L2 - Not reported	DA - Not reported; QA - Not reported	Narrative/descriptive summary only
Ndegwa, 2010	No definition	NR	Protocol not mentioned; Research question clearly reported	Searched more than one database; Searched published and grey literature; Search strategy clearly reported; Yes, references scanned; Contacting authors not reported	Limited by date; Limited by language; No limit on study design	L1 - one reviewer only; L2 - One reviewer and one verifier	DA - Not reported; QA - Not reported	Narrative/descriptive summary only
O' Meara, 2001	Reviews in Health Technology Assessment are termed systematic when the account of the search appraisal and synthesis methods would, in theory, permit the replication of the	11 months	Protocol not mentioned; Research question clearly reported	Searched more than one database; Searched published and grey literature; Search strategy clearly reported; Yes, references scanned; Yes, authors contacted	No Date limit; Limited by language; Limited by study design	L1 - two independent reviewers; L2 - Two independent reviewers	DA - One reviewer and one verifier; QA- Two independent reviewers	Meta-analysis and narrative summary

	review by others. Rapid reviews are completed in a limited time to inform the appraisal and guideline development processes managed by NICE.							
Obyn, 2008	No definition	NR	Protocol not mentioned; Research question clearly reported	Used a previous review(s) as starting point; Searched published and grey literature; Search strategy clearly reported; Yes, references scanned; Contacting authors not reported	Limited by date; Limited by language; No limit on study design	L1 - Done but unclear number of reviewers; L2 - Done but unclear number of reviewers	DA - Done but unclear number of reviewers; QA - Done but unclear number of reviewers	Narrative/descriptive summary only
Phillipson, 2012	This evidence check review was produced using the evidence check methodology in response to specific questions from the commissioning agency. It is not necessarily a comprehensive review of all literature relating to the topic area.	NR	Protocol not mentioned; Research question clearly reported	Searched more than one database; Searched published and grey literature; Search strategy clearly reported; Yes, references scanned; Contacting authors not reported	Limited by date; Language limit unclear/not reported; Study design unclear/not reported	L1 - Done but unclear number of reviewers; L2 - Done but unclear number of reviewers	DA - Done but unclear number of reviewers; QA - Done but unclear number of reviewers	Narrative/descriptive summary only
Rissel, 2012	This evidence check review was produced using the evidence check methodology in response to specific questions from the commissioning agency. It is not necessarily a comprehensive review of all literature relating to the topic area.	NR	Protocol not mentioned; Research question clearly reported	Searched more than one database; Searched published and grey literature; Search strategy clearly reported; Yes, references scanned; Contacting authors not reported	Limited by date; Limited by language; Study design unclear/not reported	L1 - Done but unclear number of reviewers; L2 - Not done	DA - Done but unclear number of reviewers; QA - Not reported	Narrative/descriptive summary only

Saborido, 2010	No definition	4 months	Protocol not mentioned; Research question clearly reported	Used a previous review(s) as starting point; Searched published and grey literature; Search strategy clearly reported; Yes, references scanned; Contacting authors not reported	No Date limit; Limited by language; No limit on study design	L1 - two independen t reviewers; L2 - Two independen t reviewers	DA - One reviewer and one verifier; QA - Not done	Narrative/d escriptive summary only
Schnell-Inderst, 2011	No definition	NR	Protocol not mentioned; Research question clearly reported	Used a previous review(s) as starting point; Searched published and grey literature; Search strategy clearly reported; Reference scanning not reported; Contacting authors not reported	Limited by date; Language limit unclear/not reported; Limited by study design	L1 - two independen t reviewers; L2 - Two independen t reviewers	DA - Two independen t reviewers; QA- Two independen t reviewers	Narrative/d escriptive summary only
Singh, 2005	No definition	NR	Protocol not mentioned; Research question clearly reported	Searched more than one database; Searched published and grey literature; Search strategy not reported; Yes, references scanned; Yes, authors contacted	Limited by date; No Language limit; No limit on study design	L1 - two independen t reviewers; L2 - Two independen t reviewers	DA - One reviewer and one verifier; QA- Two independen t reviewers	Narrative/d escriptive summary only
Singh, 2006	No definition	NR	Protocol not mentioned; Research question clearly reported	Searched more than one database; Searched published and grey literature; Search strategy clearly reported; Yes, references scanned; Yes, authors contacted	Limited by date; No Language limit; No limit on study design	L1 - two independen t reviewers; L2 - Two independen t reviewers	DA - One reviewer and one verifier; QA - Not reported	Clear reasons for not doing meta-analysis

Singh, 2006	No definition	3 weeks	Protocol not mentioned; Research question clearly reported	Searched more than one database; Searched published and grey literature; Search strategy clearly reported; Yes, references scanned; Yes, authors contacted	Limited by date; No Language limit; No limit on study design	L1 - one reviewer only; L2 - One reviewer only	DA - One reviewer only; QA - One reviewer only	Clear reasons for not doing meta-analysis
Smith, 2013	Rapid structured reviews are used to summarize and synthesis research findings within the constraints of a given timetable and resources and differ from systematic review in relation to the extensiveness of the literature search and methods used to undertake the analysis	NR	Protocol not mentioned; Research question clearly reported	Searched more than one database; Searched published and grey literature; Search strategy clearly reported; Yes, references scanned; Yes, authors contacted	Limited by date; Limited by language; Limited by study design	L1 - one reviewer only; L2 - Done but unclear number of reviewers	DA - Not reported; QA - Done but unclear number of reviewers	Narrative/descriptive summary only
Stordeur, 2009	No definition	NR	Protocol not mentioned; Research question clearly reported	Used a previous review(s) as starting point; Searched published and grey literature; Search strategy clearly reported; Yes, references scanned; Contacting authors not reported	Limited by date; No Language limit; No limit on study design	L1 - two independent reviewers; L2 - Two independent reviewers	DA - Done but unclear number of reviewers; QA - One reviewer only	Narrative/descriptive summary only
Sutton, 2011	Rapid reviews, by their very nature, are constrained by time, and this impact on the completeness of searching undertaken	NR	Protocol not mentioned; Research question clearly reported	Searched more than one database; Searched published sources only; Search strategy clearly reported;	Limited by date; Limited by language; Limited by study design	L1 - Done but unclear number of reviewers; L2 - Done but unclear	DA - Not reported; QA - Not reported	Narrative/descriptive summary only

	and the depth of formal quality assessment. Nonetheless, this type of review can provide a general indication of the overall quality and direction of effect reported in the literature.			Yes, references scanned; Contacting authors not reported		number of reviewers		
Thavaneswaran, 2009	A rapid systematic review in which the methodology has been limited in one or more areas to shorten the timeline for its completion. This rapid review is a limited evidence-based assessment that is based on a simple systematic search of studies published in the peer reviewed literature. As a result, this rapid review may be used to inform certain questions on the specific review topic.	NR	Protocol not mentioned; Research question clearly reported	Searched more than one database; Searched published and grey literature; Search strategy clearly reported; No references scanned; No authors contacted	Limited by date; Limited by language; Limited by study design	L1 - two independent reviewers; L2 - Two independent reviewers	DA - One reviewer and one verifier; QA - One reviewer and one verifier	Clear reasons for not doing meta-analysis
The Medical Advisory Secretariat, 2010	No definition	NR	Protocol not mentioned; Research question clearly reported	Database search not reported; No Grey literature mentioned; Search strategy unclear; Yes, references scanned; Contacting authors not reported	Data limit unclear/not reported; Language limit unclear/not reported; Study design unclear/not reported	L1 - not reported; L2 - Not reported	DA - Not reported; QA - Not reported	Narrative/descriptive summary only
Thigpen, 2012	The Rapid Synthesis and Translation Program is	10-12 months	Protocol not mentioned; Research	Database search not reported; No Grey literature	Data limit unclear/not reported;	L1 - Done but unclear number of	DA - Not reported; QA - Not	Unclear/limited data synthesis

	collaborative and by using Lomas' exchange model of knowledge transfer bridges the gap between research and practice by keeping researchers and practitioners involved from the beginning. The RSTP borrows from other policy and science synthesis and translation literature much of which is related to knowledge transfer includes processes or components related to translation.		question clearly reported	mentioned; Search strategy not reported; Reference scanning not reported; Contacting authors not reported	Language limit unclear/not reported; Study design unclear/not reported	reviewers; L2 - Done but unclear number of reviewers	reported	methods
Tripney, 2011	No definition	6 months	Yes, protocol mentioned; Research question clearly reported	Searched more than one database; Searched published and grey literature; Search strategy clearly reported; Yes, references scanned; Yes, authors contacted	Limited by date; Limited by language; No limit on study design	L1 - one reviewer only; L2 - One reviewer and one verifier	DA - Two independen t reviewers; QA- Two independen t reviewers	Unclear/li mited data synthesis methods
Tsakonas, 2008	HTIS responses are based on a limited literature search and are not comprehensive, systematic reviews. The intent is to provide a list of sources and a summary of the best evidence on the topic that CADTH could identify using all reasonable efforts	NR	Protocol not mentioned; Research question clearly reported	Searched more than one database; Searched published and grey literature; Search strategy not reported; Reference scanning not reported; Contacting authors not reported	Limited by date; Limited by language; Limited by study design	L1 - two independen t reviewers; L2 - Not reported	DA - Not reported; QA - Done but unclear number of reviewers	Narrative/d escriptive summary only

	within the time allowed. HTIS responses should be considered along with other types of information and health care considerations. The information included in this response is not intended to replace professional medical advice, nor should it be construed as a recommendation for or against the use of a particular health technology.							
Van Brabandt, 2009	No definition	NR	Protocol not mentioned; Research question clearly reported	Searched more than one database; Searched published and grey literature; Search strategy clearly reported; Reference scanning not reported; Yes, authors contacted	Data limit unclear/not reported; Language limit unclear/not reported; Study design unclear/not reported	L1 - not reported; L2 - Not reported	DA - Done but unclear number of reviewers; QA - Done but unclear number of reviewers	Narrative/descriptive summary only
Van Branadt, 2008	No definition	NR	Protocol not mentioned; Research question clearly reported	Used a previous review(s) as starting point; Searched published and grey literature; Search strategy clearly reported; No references scanned; No authors contacted	No Date limit; No Language limit; No limit on study design	L1 - Done but unclear number of reviewers; L2 - Done but unclear number of reviewers	DA - Done but unclear number of reviewers; QA - Not reported	Narrative/descriptive summary only
Vlayen, 2006	No definition	NR	Protocol not mentioned; Research question	Searched more than one database; Searched published and grey literature;	Limited by date; No Language limit; Limited by	L1 - one reviewer only; L2 - Done but	DA - Done but unclear number of reviewers;	Meta-analysis and narrative

			clearly reported	Search strategy clearly reported; Reference scanning not reported; Yes, authors contacted	study design	unclear number of reviewers	QA - One reviewer and one verifier	summary
WHO, 2006	No definition	6 months	Protocol not mentioned; Research question clearly reported	Searched more than one database; Searched published sources only; Search strategy clearly reported; Yes, references scanned; Contacting authors not reported	Limited by date; Language limit unclear/not reported; Limited by study design	L1 - two independen t reviewers; L2 - Two independen t reviewers	DA - One reviewer and one verifier; QA- Two independen t reviewers	Narrative/d escriptive summary only
York, 2011	The Rapid Evidence Assessment of the Literature approach differs from a conventional systematic literature review in that a REAL provides an evidence-based, systematic ‘‘snapshot’’ of the available literature.	NR	Protocol not mentioned; Research question clearly reported	Searched more than one database; Searched published and grey literature; Search strategy clearly reported; Reference scanning not reported; Contacting authors not reported	No Date limit; Limited by language; No limit on study design	L1 - More than two reviewers; L2 - Done but unclear number of reviewers	DA - Done but unclear number of reviewers; QA - More than two reviewers	Narrative/d escriptive summary only
		8-12 weeks	Protocol not mentioned; Research question clearly reported	Searched more than one database; Searched published sources only; Search strategy clearly reported; No references scanned; No authors contacted	Data limit unclear/not reported; Language limit unclear/not reported; Study design unclear/not reported	L1 - Done but unclear number of reviewers; L2 - Done but unclear number of reviewers	DA - Done but unclear number of reviewers; QA - Not reported	Narrative/d escriptive summary only
